# Monitoring phosphorus content in winter wheat using feature fusion and feature selection from UAV remote sensing imagery

**DOI:** 10.3389/fpls.2026.1851992

**Published:** 2026-05-21

**Authors:** YiMing Su, JingXia Wang, YunMa Yang, JunFang Yang, Jing Zhang, HuiMin Yang, YongKang Duan, ShuoYang Han, Kang Yu, ShaoHui Huang, LiangLiang Jia

**Affiliations:** 1 Institute of Agricultural Resources and Environment, Hebei Academy of Agriculture and Forestry Sciences, Shijiazhuang, China; 2 Precision Agriculture Laboratory, School of Life Sciences, Technical University of Munich, Freising, Germany

**Keywords:** feature selection, multi-source feature fusion, plant phosphorus content, UAV images, winter wheat

## Abstract

The rapid and accurate quantification of plant phosphorus (P) content is essential for the real-time assessment of crop P status and improvement of P fertilizer use efficiency. However, non-destructive and rapid approaches for P monitoring are limited. In this study, the feasibility of monitoring plant phosphorus content (PPC) in winter wheat was evaluated through multi-source feature fusion of unmanned aerial vehicle (UAV) imagery based on a long-term field experiment with five P treatments. Multiple spectral features, including color indices (CIs), fractional vegetation cover (FVC), vegetation indices (VIs), texture features (TFs) and texture indices (TIs), were extracted from UAV RGB and multispectral images. Sensitive spectral features were systematically screened using Pearson correlation analysis, random forest (RF) importance ranking, and the Relief algorithm. Selected features were then fed into three machine learning models, RF, support vector machine (SVM), and k-nearest neighbor (KNN) to predict PPC. The results showed that GRI, VARI, MGRVI, TGI, NDRE, and CIred edge were highly correlated with PPC at the maturity stage (r = 0.96). Both TFs and TIs demonstrated stronger correlations with PPC at the 750 and 840 nm bands, with most TIs outperforming TFs, confirming the feasibility of spectral-based PPC estimation. Based on the selected input variables including DTI (450-Ent, 750-Mea), 840-Mea, and RVI, the SVM model achieved the best performance (R^2^c=0.94, RMSEc=0.29, RPDc=4.03; R^2^v=0.92, RMSEv=0.36, RPDv=3.48). These results highlight the potential of combining VIs, TFs, and TIs features for training machine learning models for PPC prediction, while the organ-level physiological explanations warrantee further investigations under controlled P gradients. This study provides data-driven insights for UAV-based monitoring of plant P nutritional status under local experimental conditions.

## 1 Introduction

As a globally pivotal staple food crop, wheat in China accounts for more than 10% of the global wheat planting area (Jiao et al., 2024), and its stable yield contributes fundamentally to securing global food supplies (Huang et al., 2025). As an essential component of plant compounds, phosphorus regulates crop yield, photosynthesis, grain formation and stress resistance, underpinning stable production (Sun et al., 2019; Xia et al., 2021). Therefore, monitoring plant phosphorus content (PPC) is fundamental to precision fertilization and food security (Cordell et al., 2009). Accordingly, the efficient and accurate determination of PPC is crucial for regulating wheat growth and improving its yield and quality.

Conventional PPC monitoring relies on manual sampling and laboratory testing, with high labor cost, destructiveness and limited spatial representativeness. Although spectral techniques have been widely applied in crop nutrient monitoring (Jiang et al., 2025; Li et al., 2025a; Zhang et al., 2025), rapid and accurate diagnosis of plant P status remains insufficient for real-time P evaluation and improved P fertilizer use efficiency (Ban et al., 2021; Li et al., 2018; Wang et al., 2025). Among different spectral diagnostic approaches, satellite remote sensing is susceptible to weather and cloud interference and is constrained by relatively low spatial resolution (Oliveira et al., 2024). In contrast, ground-based platforms suffer from limited spatial coverage, low operational efficiency, and difficulty in dynamic monitoring. Benefiting from high spatiotemporal resolution and operational flexibility, unmanned aerial vehicle (UAV) remote sensing enables the rapid acquisition of farmland information and has been increasingly applied in crop nutrient parameter monitoring (Li et al., 2025b; Qiao et al., 2020; Zhang et al., 2022).

In UAV-based nutrient monitoring, color features derived from Red, Green, and Blue (RGB) imagery can directly reflect the morphological changes induced by nutrient stress but are highly sensitive to illumination and shadow conditions (Cao et al., 2021; Gan et al., 2023). To alleviate this limitation, Color Indices (CIs) based on RGB channels have been developed to enhance vegetation-related features (Li et al., 2019). In contrast, multispectral imagery provides richer band information and exhibits stronger responsiveness to crop spectral variations (Song et al., 2025). Numerous studies have employed Vegetation Indices (VIs) to quantify vegetation information and estimate Fractional Vegetation Cover (FVC) (Qiao et al., 2024; Zhang et al., 2025). However, spectral saturation frequently occurs under conditions of high crop coverage (Feng et al., 2025). Consequently, texture features (TFs) have been introduced to characterize canopy structural changes and compensate for the limitations of VIs (Meng et al., 2025; Qiao et al., 2024). Furthermore, Texture Indices (TIs) derived from multi-band TFs enhance the responsiveness of texture features to crop parameters and improve monitoring accuracy (Li et al., 2019). Since models using single spectral features are prone to external interference, insufficient information and weak generalization, multi-source feature fusion serves as a key strategy to improve nutrient monitoring accuracy (Li et al., 2025a; Zhang et al., 2024a). Nevertheless, studies integrating UAV RGB and multispectral data for monitoring winter wheat PPC remain limited, highlighting the need for further investigation.

In addition, UAV-based multi-source remote sensing integrates spectral, textural, and structural features. However, the resulting high dimensionality inevitably introduces feature redundancy, which can affect the model generalization performance (Shao et al., 2025; Wei et al., 2025). Therefore, feature selection is a critical step for improving model accuracy by retaining key relevant variables while eliminating redundant or noisy information (Xiao et al., 2025). Moreover, owing to its simple computation and explicit physical interpretability, Pearson correlation analysis has been widely applied to spectral feature selection (Li et al., 2018; Zhang et al., 2025). Meanwhile, Random Forest (RF) feature importance ranking is increasingly used to quantify feature contributions and serves as an effective method for high-dimensional remote sensing feature selection (Prado et al., 2019). In contrast, the Relief algorithm iteratively updates feature weights and retains variables with positive contributions (Zang et al., 2024; Zhou et al., 2023). Previous studies have confirmed that feature selection algorithms can enhance model accuracy through dimensionality reduction and optimized variable selection. However, their application in PPC estimation remains insufficiently explored.

Machine learning algorithms have been widely employed in crop nutrient monitoring because of their strong capability to model nonlinear relationships (Mali et al., 2025; Xiao et al., 2025; Zhu et al., 2025). Such applications often rely on multi-source sensor data, generating datasets that contain not only rich spectral information, but also texture and thermal infrared features (Qiao et al., 2024; Zhang et al., 2024a). Existing studies have shown that the RF algorithm performs well in handling high-dimensional data and mitigates overfitting effects (Fu et al., 2025; Gan et al., 2023). In comparison, the support vector machine (SVM) algorithm has been reported to outperform simple linear models in terms of generalization ability under small-sample conditions (Feng et al., 2024). In addition, the k-nearest neighbor (KNN) model is noted for its rapid response capability in crop parameter monitoring when sample sizes are limited (Teshome et al., 2023). Overall, machine learning algorithms enable the efficient and accurate estimation of crop nutrient parameters, but their robustness and generalization performance require further validation.

Furthermore, crop P content significantly influences spectral characteristics (Wang and Bai, 2006). P deficiency induces physiological, metabolic, morphological and structural changes in plants, altering leaf light absorption and reflection traits and causing distinct spectral band differences (Al-Abbas et al., 1974; Milton et al., 1991). Notably, P is involved in critical plant physiological processes including photosynthesis, respiration, and carbohydrate translocation (Ma et al., 2022; Shu et al., 2023). Its deficiency inhibits chloroplast development and interferes with the photosynthetic process, resulting in narrow, thin leaves and loosely arranged mesophyll cells (Li et al., 2022). It also impedes the translocation of photosynthetic products, suppresses the activity of chlorophyll synthase, and ultimately reduces photosynthetic efficiency (Qiu and Israel, 1992). Essentially, these spectral variations directly reflect alterations in plant biochemical components and structural parameters caused by P deficiency (Li et al., 2022). Previous studies have reported species-specific spectral sensitivities to crop P content. For example, the 713 nm band was sensitive to rubber leaf P content, attributed to the close relationship between red edge reflectance and chlorophyll content-closely linked to P levels (Guo et al., 2018). For winter wheat, strong correlations between leaf P content and spectral reflectance have been observed at 630, 720, and 780 nm (Wang et al., 2025). In contrast, the sensitive spectral range for rapeseed P content was 730–1300 nm (Li et al., 2016), whereas sensitive bands for rice were 670, 706, 722, and 846 nm (Ban et al., 2021). Collectively, sensitive bands for crop P content estimation are distributed across the visible to near-infrared spectra, yet previous studies have failed to reach a consistent consensus. Although P is an essential macronutrient for crops, the relationship between P and spectral response remains unclear due to its complex effects on pigment synthesis, substance accumulation, and cell structure, requiring further investigation. Accordingly, this study aimed to (1) investigate the feasibility of multi-source feature fusion for monitoring winter wheat PPC, (2) evaluate the feasibility of different feature selection methods for PPC estimation, and (3) assess the effects of various machine learning algorithms on estimation accuracy. Meanwhile, this study clarified three clear contributions: targeting winter wheat P monitoring, integrating multiple spectral features and texture indices as multi-source remote sensing features for P inversion, and identifying the optimal feature combination with reasonable literature-supported physiological interpretation.

## 2 Materials and methods

### 2.1 Experimental design

**Figure 1:**
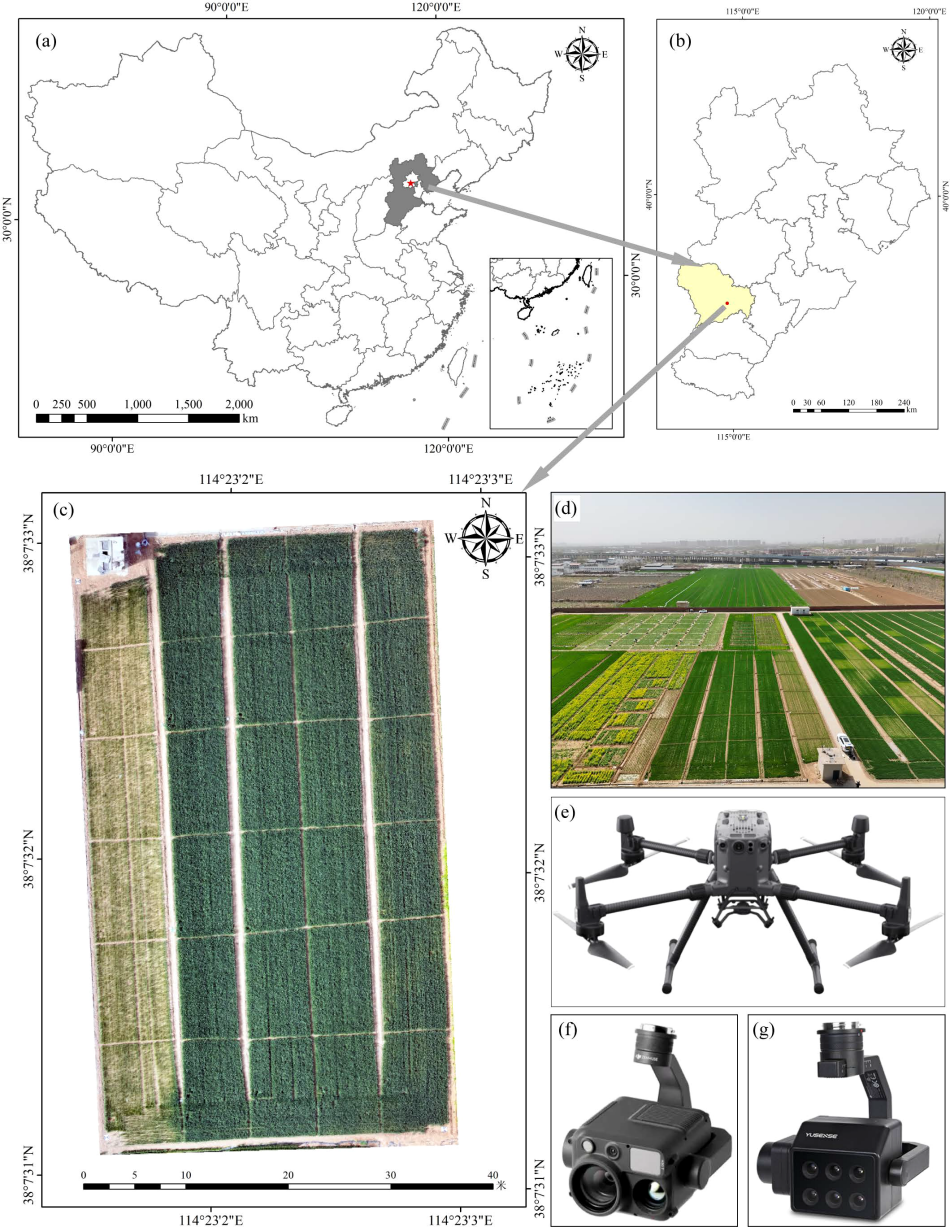
Overview of the study area, UAV and sensors: **(a-d)** Location of the test area; **(e)** UAV DJM300 RTK; **(f)** H30T Camera; **(g)** MS600 Multispectral Camera.

The experiment was performed at the Comprehensive Experimental Station of the Hebei Academy of Agriculture and Forestry, located in Dahe Town, Luquan District, Shijiazhuang City, Hebei Province, China (38°07′N, 114°23′E). The region has a humid continental monsoon climate. Annual precipitation ranges from 300 to 600 mm, 70-80% of which falls between June and September. The annual mean temperature is 14.3 °C, with a frost-free period of 198 d. This study was based on a long-term field P management experiment established in October 2008 with a 17-year implementation history under a wheat-maize rotation system. Five annual P fertilizer application rates, expressed as P_2_O_5_, were applied: P0 (0 kg/ha), P1 (75 kg/ha), P2 (120 kg/ha), P3 (165 kg/ha), and P4 (210 kg/ha). Three replications were arranged for each treatment, leading to 15 plots with a size of 120 m² per plot. (**Figures 1a–d**). P fertilizer was applied once during the wheat growing season, whereas only nitrogen and potassium fertilizers were applied during the maize season. In the present study, the winter wheat cultivar Zhongmai 6032 was sown in October 2024 and harvested in June 2025. UAV remote sensing data acquisition and field sampling were conducted from March to June 2025. All other field management practices followed local farmers’ conventional management.

### 2.2 Multispectral data acquisition and processing of UAV

**Figure 2:**
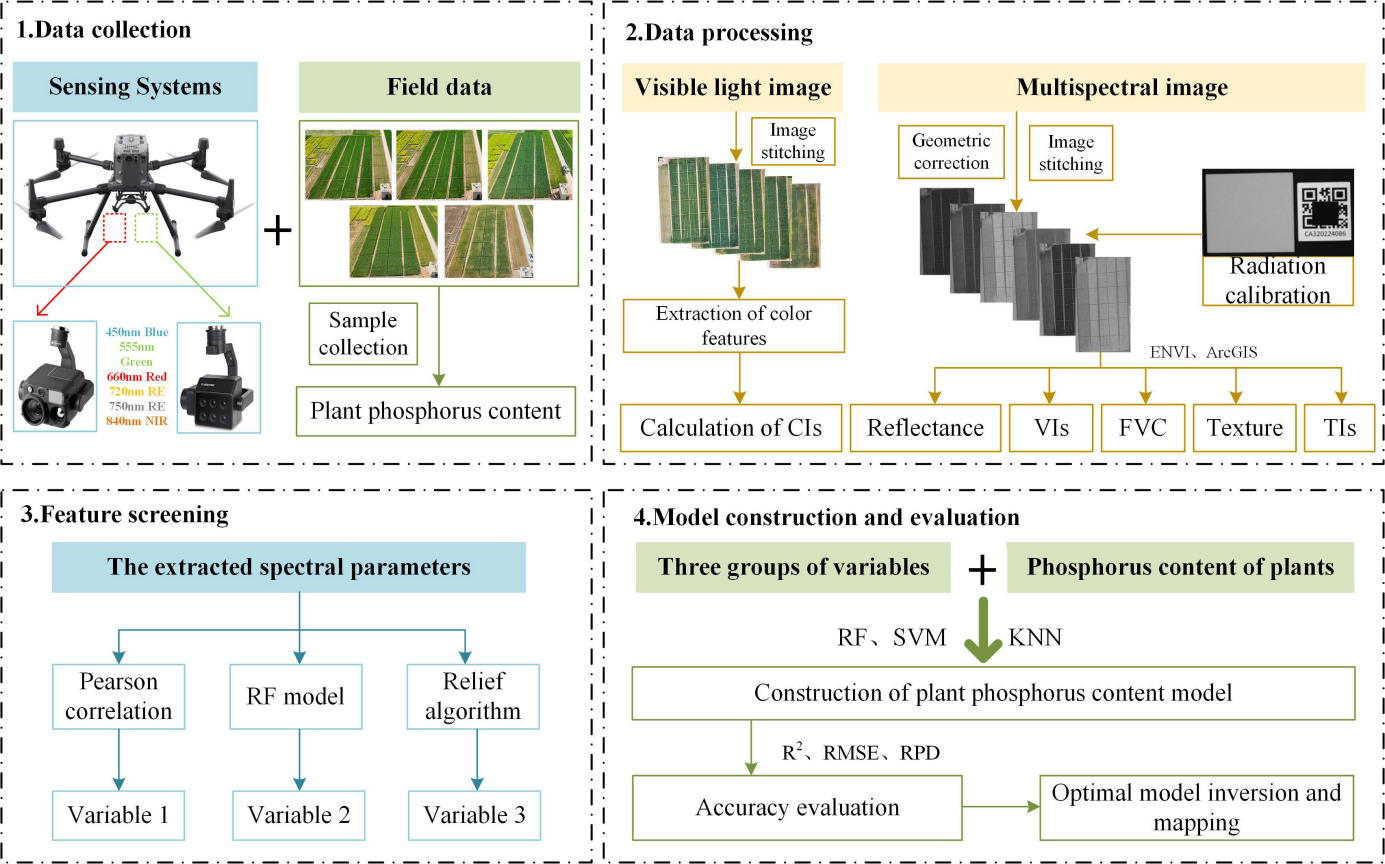
Technical roadmap.

UAV images were acquired using a DJ M300 RTK platform (**Figure 1e**) equipped with an H30T camera and an MS600 multispectral camera to obtain RGB and six-band multispectral images, respectively (**Figures 1f, g**). The spectral parameters of the MS600 multispectral camera are presented in **Table 1**. Flight missions were conducted using DJI Pilot software, with image acquisition performed between 10:00 and 12:00 local time during the jointing, booting, heading, filling, and maturity stages of winter wheat. The UAV was flown at an altitude of 50 m, and both the forward and lateral overlap ratios were set to 85%. Ground control points (GCPs) were used for precise co-registration of UAV RGB and multispectral images. RGB imagery was preprocessed in Pix4D Mapper to implement lens distortion correction, illumination normalization and mosaicking for orthomosaic generation. Multispectral data underwent geometric correction and radiometric calibration in Pix4D Mapper. Combined with camera radiometric parameters and standard gray board images, absolute reflectance calibration was conducted to standardize radiation information. Band fusion of multispectral imagery was completed in ENVI Classic 5.3. All images were imported into ENVI 5.3 to delineate regions of interest (ROIs) and extract digital number (DN) values and surface reflectance. For ROI delineation, each plot was inward buffered by 0.5-1.0 m to eliminate disturbances from terrain relief, shadows and weeds. A unified coordinate system and vector-assisted ROI mapping were used to reduce manual errors. The overall research framework of this study is shown in **Figure 2**.

**Table 1:** MS600 Multispectral camera six-band parameters.

Band number	Center wavelength(nm)	Band name	Band width (nm)
B1	450	Blue	30
B2	555	Green	27
B3	660	Red	22
B4	720	Red edge	10
B5	750	Red edge	10
B6	840	Near infrared	30

### 2.3 Determination of winter wheat PPC

During the jointing, booting, heading, filling, and maturity stages of winter wheat, 15 cm-long double-row plant samples were collected from representative plots exhibiting uniform growth and immediately sealed in bags. Field sampling was conducted synchronously with UAV image acquisition. After separating the stems and leaves, the samples were subjected to enzyme deactivation by heating at 105°C for 30 min, followed by oven drying at 80°C to a constant weight. After sulfuric acid-hydrogen peroxide digestion, PPC was determined by the molybdenum antimony anti-colorimetric method using an ultraviolet-visible (UV-Vis) spectrophotometer.

### 2.4 Calculation of CIs and VIs

Based on previous studies, 14 commonly used CIs and 12 VIs were selected to monitor winter wheat PPC (**Table 2**). Among these, CIs were calculated from the DN values and normalized values of the RGB bands. The VIs primarily consist of visible, red edge, and near-infrared (NIR) bands, integrating plant spectral absorption, reflectance characteristics, and background effects. This integration enabled VIs to more effectively capture variations in crop nutrient status.

**Table 2:** Calculation formulas and references of CIs and VIs.

Data type	Index	Calculation Formula	Reference
Color Index	R(Red)	-	(Woebbecke et al, 1995)
	G(Green)	-	
	B(Blue)	-	
	Normalized red band(r)	*R/(R+G+B)*	
	Normalized green band(g)	*G/(R+G+B)*	
	Normalized blue band(b)	*B/(R+G+B)*	
	Blue/green pigment index(BGI)	*b/g*	(Zhang et al, 2015)
	Blue/red pigment index(BRI)	*b/r*	
	green/red pigment index(GRI)	*g/r*	(Gamon and Surfus, 1999)
	Green red vegetation index(GRVI)	*(g-r)/(g+r)*	(Tucker, 1979)
	Visible atmospherically resistant index(VARI)	*(g-r)/(g+r-b)*	(Gitelson et al, 1996)
	Modified green red vegetation index(MGRVI)	*(g^2^-r^2^)/(g^2^+r^2^)*	(Bendig et al, 2015123)
	Red-green-blue vegetation index(RGBVI)	(g^2^-r*b)/(g^2^+r*b)	
	Triangular greenness index(TGI)	-0.5*[190*(r-g)-120*(r+b)]	(Hunt et al, 2013)
Vegetation Index	Normalized difference vegetation index (NDVI)	*(R_NIR_-R_RED_)/(R_NIR_+R_RED_)*	(Rouse et al, 1973)
	Green Normalized Difference Vegetation Index(GNDVI)	*(R_NIR_-R_GREEN_)/(R_NIR_+R_GREEN_)*	(Shaver et al, 2006)
	Blue Normalized Difference Vegetation Index(BNDVI)	*(R_NIR_-R_BLUE_)/(R_NIR_+R_BLUE_)*	(Van and Price, 2015)
	Normalized Difference Red Edge Index(NDRE)	*(R_NIR_-R_RE_)/(R_NIR_+R_RE_)*	(Gitelson et al, 1996)
	The MERIS terrestrial chlorophyll index (MTCI)	*(R_NIR_-R_RE_)/(R_RE_-R_RED_)*	(Dash and Curran, 2004)
	Different vegetation index (DVI)	*R_NIR_-R_RED_*	(Naito et al, 2017)
	Ratio vegetation index (RVI)	*R_NIR_/R_RED_*	(Liang et al, 2013)
	Green Ratio Vegetation Index(GRVI)	*R_NIR_/R_GREEN_*	(Motohka et al, 2010)
	Red edge chlorophyll index (CI_red-edge_)	*R_NIR_/R_RE_-1*	(Gitelson et al, 1996)
	Enhanced Vegetation Index(EVI)	*2.5[(R_NIR_-R_RED_)/(R_NIR_+6R_RED_-7.5R_BLUE_+1)]*	(Prabhakara et al, 2015)
	Optimized soil-adjusted vegetation index (OSAVI)	*(1 + 0.16)(R_NIR_-R_RED_)/(R_NIR_+R_RED_+0.16)*	(Rondeaux et al, 1996)
	Excess Green Index(ExG)	*2(R_GREEN_-R_RED_-R_BLUE_)/(R_GREEN_+R_RED_+R_BLUE_)*	(Rasmussen et al, 2016)

R, G, and B represent the red, green, and blue digital number (DN) values of RGB pixels, respectively; r, g, and b represent the red, green, and blue values derived after the normalization of pixel DN values, respectively; *R_RED_*, *R_GREEN_*, *R_BLUE_*, *R_RE_*represent the reflectance of multispectral red, green, blue, red edge, and NIR bands, respectively.

### 2.5 Extraction of FVC

In this study, the FVC within the experimental area was estimated using a dimidiate pixel model based on confidence intervals. Specifically, the NDVI was first calculated for each plot, after which the confidence intervals for bare soil and fully vegetated areas were determined from the statistical distribution of NDVI values. The minimum and maximum NDVI values within these intervals were defined as the NDVI values for bare soil and fully vegetated surfaces, respectively. The 95% confidence interval served as the vegetation endmember threshold, and the 5% interval as the soil endmember threshold for pixel classification and decomposition. FVC for the entire experimental area was calculated using the following Equation 1 (Carlson and Ripley, 1997):

(1) $$FVC=\frac{NDVI-NDVI_{soil}}{NDVI_{veg}-NDVI_{soil}}$$

where NDVI denotes the NDVI value of the current pixel, NDVI_soil_ represents the NDVI value of pure bare soil pixels, and NDVI_veg_ refers to the NDVI value of pure vegetated pixels.

### 2.6 Extraction and calculation of TFs

TFs were extracted from six-band multispectral images using the gray level co-occurrence matrix (GLCM). The original gray values were quantized to 64 levels, and a 3×3 pixel moving window was used for statistical calculation. GLCM metrics were computed in four directions (0°, 45°, 90°, and 135°) with a 1-pixel offset, and the outputs from all directions were averaged (Liu et al., 2023; Niu et al., 2025). Eight TFs were generated for each band, yielding 48 features in total: mean (MEA), variance (VAR), homogeneity (HOM), contrast (CON), dissimilarity (DIS), entropy (ENT), second moment (SEM), and correlation (COR). To further explore the contribution of UAV multispectral texture features (TFs) to winter wheat PPC monitoring, three texture indices (TIs) were constructed via TF transformation: normalized difference texture index (NDTI), ratio texture index (RTI), and difference texture index (DTI). The corresponding Equations 2–4 are as follows (Hang et al., 2021):

(2) $$NDTI=\frac{T_{1}-T_{2}}{T_{1}+T_{2}}$$

(3) $$RTI=T_{1}/T_{2}$$

(4) $$DTI=T_{1}-T_{2}$$

where T_1_ and T_2_ denote TFs from random bands.

### 2.7 Spectral feature selection

Three feature selection methods, namely Pearson correlation analysis, RF importance ranking, and the Relief algorithm, were employed to screen spectral features, and the selected variables were subsequently used to construct winter wheat PPC monitoring models. Pearson correlation-based selection involved analyzing the relationships between individual spectral parameters and PPC, with variables exhibiting strong correlation coefficients retained for modeling (Li et al., 2018). To identify the optimal feature quantity, we systematically tested multiple feature subset sizes, including the top 5, 10, 15, 20, and 25 features. The top 10 features were ultimately selected due to their stable and superior validation performance, which effectively balanced model accuracy and computational complexity. The RF importance ranking was applied to order spectral features according to their contributions to the model, and the top 10 features with the highest importance scores were selected (Hastie et al., 2009). The Relief algorithm calculated feature weights through iterative updating, and the top 10 spectral features were selected in descending order of weight (Kira and Rendell, 1992). To avoid information leakage, all feature selection procedures (Pearson correlation analysis, RF importance ranking, and Relief algorithm) were performed solely on the training set. The validation set was completely excluded throughout the feature selection process. This strict operation prevents validation data from participating in correlation calculation and feature importance evaluation, thereby eliminating the overestimation of model performance.

### 2.8 Model construction and evaluation

Three machine learning algorithms, including RF, SVM, and KNN, were employed to construct winter wheat PPC monitoring models. The dataset was randomly split into training and test sets at a 3:1 ratio. All input data were standardized to eliminate dimensional effects. Five-fold cross-validation was conducted on the training set for hyperparameter optimization and model selection. The specific hyperparameter settings are as follows:

1. RF: The optimized parameters consisted of the number of decision trees (ntrees = 100), minimum leaf size (MinLeafSize = 5), and maximum number of splits (MaxNumSplits = 20). The number of randomly selected predictors (mtry) was adjusted according to different input variables;
2. SVM: The radial basis function (RBF) kernel was adopted, with the optimal penalty coefficient C = 10 and kernel parameter γ=0.1;
3. KNN: Z-score standardization was applied to input data, and the optimal number of neighbors K = 7 was determined by five-fold cross-validation.

The model performance was evaluated using three commonly adopted indicators, including coefficient of determination (R^2^), root mean square error (RMSE), and relative prediction deviation (RPD). The corresponding calculation Equations 5–8 are provided below (Hastie et al., 2009).

(5) $$R^{2}=1-\frac{\sum_{i=1}^{n}{\left(Y_{i}-Y_{i}^{\prime }\right)}^{2}}{\sum_{i=1}^{n}{\left(Y_{i}-{\bar{Y}}_{i}\right)}^{2}}$$

(6) $$RMSE=\sqrt{\frac{1}{n}\sum_{i=1}^{n}{\left(Y_{i}-Y_{i}^{\prime }\right)}^{2}}$$

(7) $$RPD=\frac{SD}{RMSE}$$

(8) $$SD=\sqrt{\frac{\sum_{i=1}^{n}{\left(Y_{i}-Y_{i}^{\prime }\right)}^{2}}{n}}$$

where n denotes the number of samples; ${\text{Y}}_{\text{i}}^{\prime }$, Y_i_, and $\bar{\text{Y}}$ represent the predicted values, observed values, and mean value of the samples, respectively; and SD is the standard deviation of the observed values.

## 3 Results

### 3.1 Variations in winter wheat PPC and spectral characteristic parameters under different P application treatments

**Figure 3:**
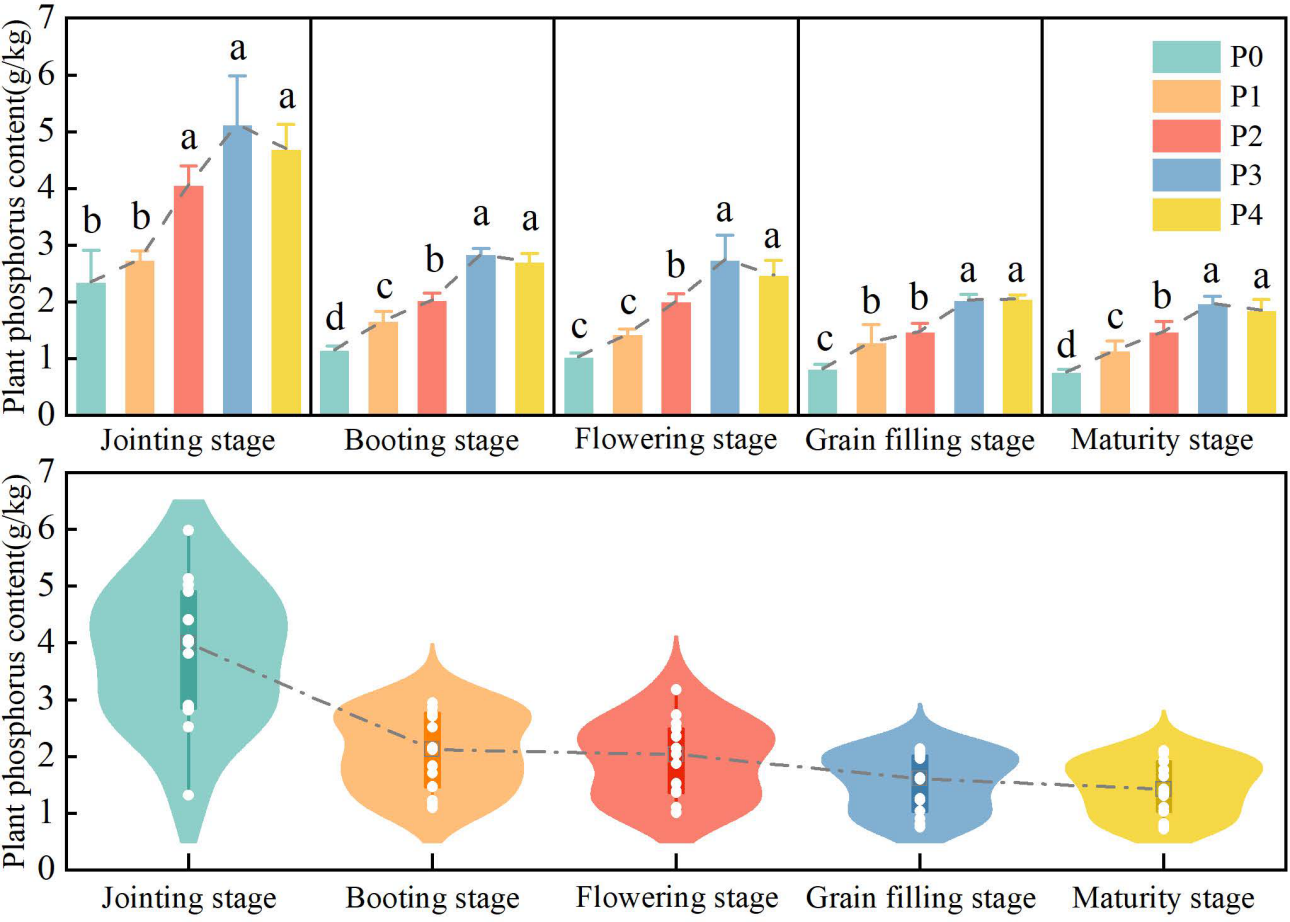
The changes of PPC in winter wheat under different phosphorus application treatments during the growth period. P0, P1, P2, P3 and P4 respectively represent five different annual phosphorus application amounts: 0, 75, 120, 165 and 210 kg/ha. Between-treatment differences were assessed via one-way ANOVA and Duncan’ s multiple range test (p< 0.05). Different lowercase letters indicate significant differences among treatments at the same growth stage.

To examine the influences of different P application treatments on winter wheat PPC, variations in PPC across growth stages under distinct P regimes were analyzed (**Figure 3**). At the jointing stage, PPC under P2, P3, and P4 was significantly higher than that under P0 and P1, with P3 exhibiting the highest mean value (5.14 g/kg) and P0 the lowest. From booting to maturity, differences in PPC among treatments gradually narrowed. P3 and P4 consistently showed significantly higher PPC than other treatments, while P0 remained at a relatively low level. Overall, both P application rate and growth stage exerted significant effects on winter wheat PPC. Treatments P3 and P4 outperformed the others, and as growth progressed, inter-treatment differences diminished alongside an overall downward trend in PPC.

**Figure 4:**
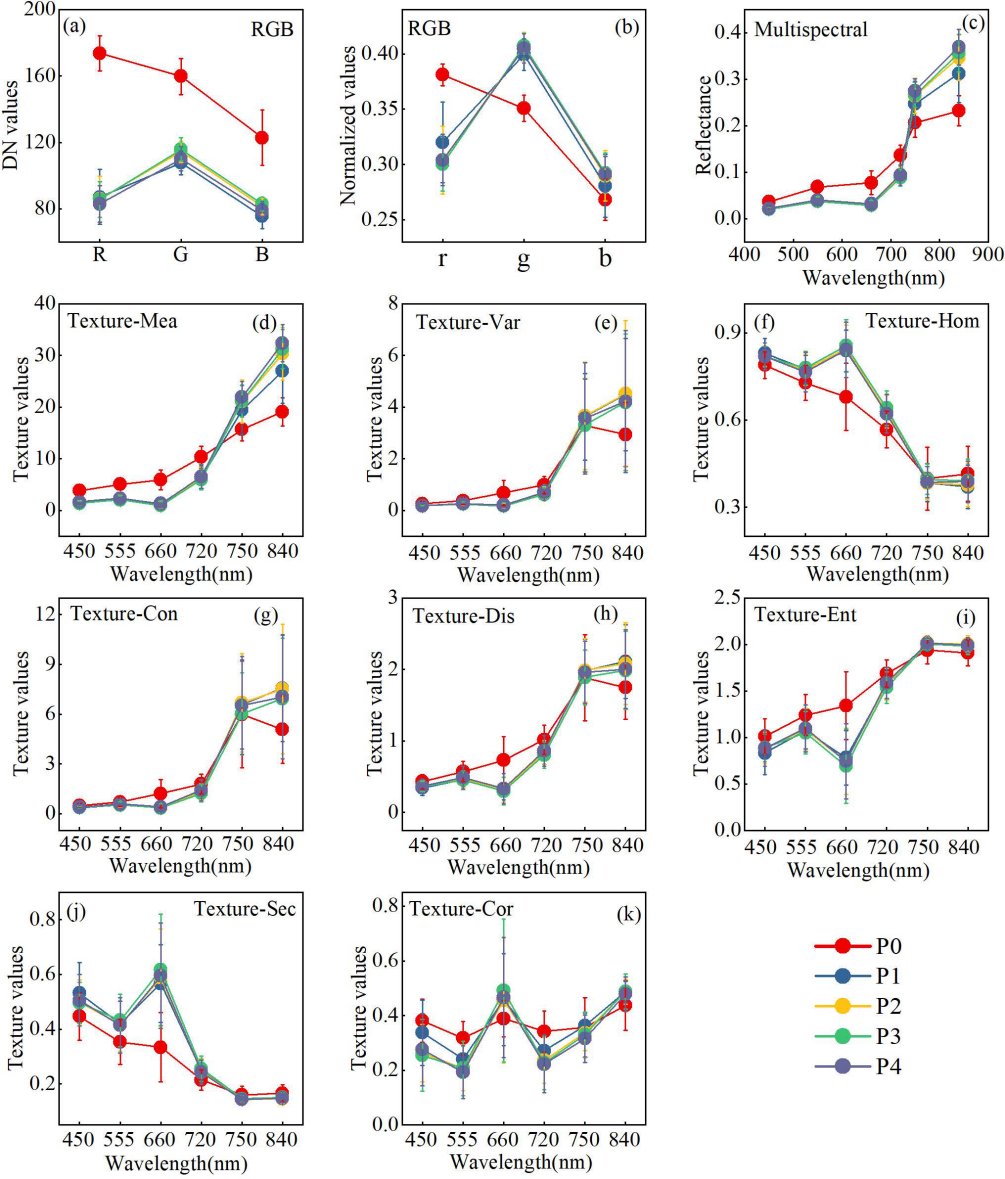
Changes in spectral characteristic parameters under different P application treatments. **(a, b)** Color features extracted from RGB images; **(c)** Reflectance extracted from multispectral images; **(d-k)** Changes in the values of eight texture features at different bands.

To further explore the impacts of P application on spectral properties, variations in RGB color features, multispectral band reflectance, and TFs were examined. For RGB DN values (**Figure 4a**), the red channel DN of P0 was significantly higher than the green and blue channels. Other treatments exhibited relatively small DN differences, with the green channel generally presenting the highest values. In terms of RGB normalized values (**Figure 4b**), P0 displayed the highest r value, whereas the g values dominated in the other treatments, with r and b values being comparable. Regarding the multispectral reflectance (**Figure 4c**), P0 exhibited higher reflectance at 450, 555, 660, and 720 nm, and lower reflectance at 750 and 840 nm than the P-applied treatments. The reflectance at 720, 750, and 840 nm increased progressively, with P2-P4 exhibiting significantly higher values than P0, indicating that P application enhanced the red edge and NIR reflectivity. For TFs (**Figures 4d–k**), Mea at 750 nm and 840 nm, Var, Con, and Dis at 840 nm, as well as Hom, Sec, and Cor at 660 nm, were significantly higher in P-applied treatments than in P0, whereas Ent at 660 nm was significantly lower. Overall, P0 differed markedly from P1-P4 in the red, red edge, and NIR bands, whereas P1-P4 exhibited similar spectral and texture feature values. These results revealed that P application significantly affected the RGB color characteristics, multispectral reflectance and texture features (TFs) of winter wheat. Red-edge and NIR band reflectance, along with their corresponding TFs, exhibited more pronounced responses to P fertilization.

### 3.2 Correlations between winter wheat PPC and spectral characteristic parameters

**Figure 5:**
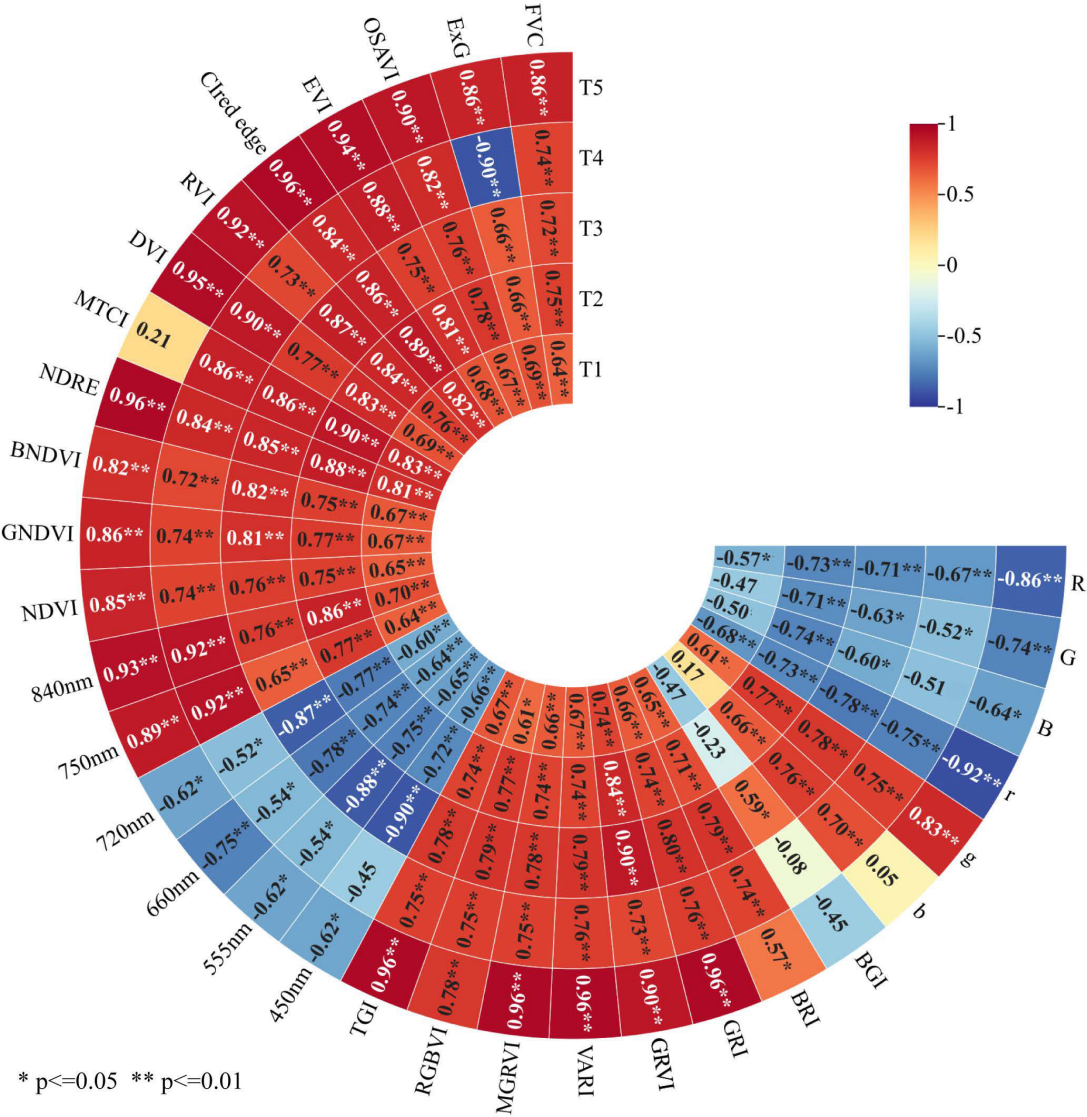
Correlation graph between PPC and spectral characteristics of winter wheat. T1, T2, T3, T4 and T5 represent the jointing stage, booting stage, flowering stage, grain filling stage and maturity stage, respectively.

To characterize the relationships between winter wheat PPC and spectral characteristics, correlations between PPC and CIs, multispectral reflectance, VIs, and FVC across different growth stages were analyzed (**Figure 5**). For CIs, R, G, B, and r showed negative correlations with PPC, whereas g and b exhibited positive correlations. Among these variables, r, R, and g displayed the strongest correlations at the T5 stage, with correlation coefficients of -0.92, -0.86, and 0.83, respectively. Overall, most CIs were positively correlated with PPC. Specifically, GRI, VARI, MGRVI, and TGI achieved the highest correlation coefficients with PPC at T5 (r = 0.96). For multispectral reflectance, bands at 450, 555, 660, and 720 nm were negatively correlated with PPC, whereas reflectance at 750 and 840 nm exhibited positive correlations. The reflectance at 840 nm showed the most significant correlation with PPC at T5 (r = 0.93), followed by the combined reflectance at 750 and 840 nm at the T4 stage (r = 0.92). Among VIs, most indices were positively correlated with PPC, with NDRE and CIred edge demonstrating the strongest correlations at T5 (r = 0.96), followed by DVI (r = 0.95). FVC was also positively correlated with PPC, with the highest correlation observed at the T5 stage (r = 0.86). In general, most spectral characteristics exhibited stronger correlations with winter wheat PPC at the T5 stage, particularly GRI, VARI, MGRVI, TGI, NDRE, and CIred edge, indicating close associations between these indices and PPC.

**Figure 6:**
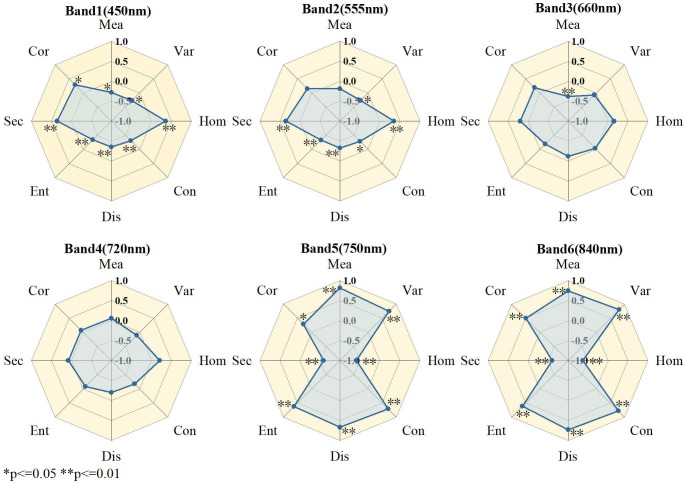
Correlation graph of PPC in winter wheat and texture characteristics in different bands.

**Figure 7:**
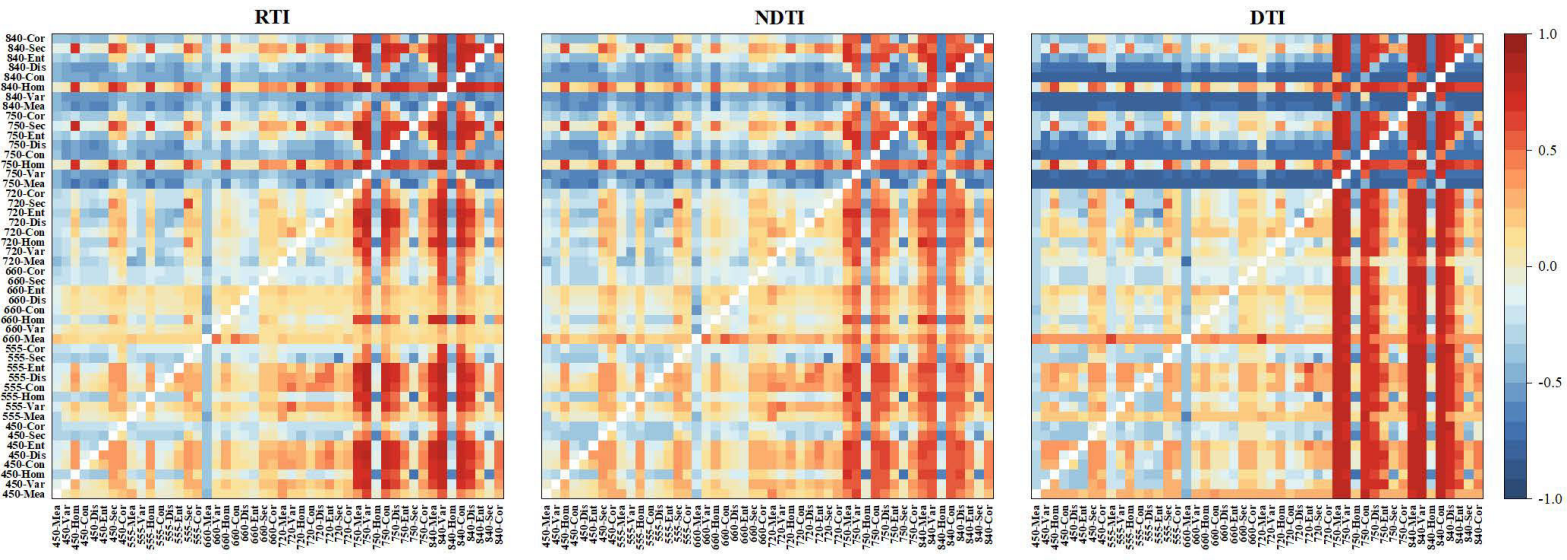
Correlation heatmap of winter wheat PPC with RTI, NDTI, and DTI. 450-Mea represents the Mean feature at the 450nm band and so on.

To further examine the relationships between TFs at different bands and winter wheat PPC, correlations between eight TFs derived from six spectral bands and PPC were analyzed (**Figure 6**). TFs at 450, 555, 660, and 720 nm exhibited relatively weak correlations with PPC, whereas most TFs at 750 and 840 nm, excluding Cor, exhibited strong correlations (r > 0.5). The Mea feature at 750 nm showed the highest correlation with PPC (r = 0.82), followed by the Var feature at 840 nm (r = 0.81). To further elucidate the relationship between TFs and PPC, three TIs were calculated, and their correlations with PPC were analyzed (**Figure 7**). Most TIs derived from TFs at 750 and 840 nm exhibited stronger correlations with PPC. Specifically, for RTI, the index constructed from 750-Hom and 840-Mea achieved the highest correlation (r = 0.84), outperforming the original TFs. For NDTI, NDTI (840-Hom, 750-Mea) reached a maximum correlation coefficient of 0.74 with PPC. For DTI, DTI (450-Ent, 750-Mea) exhibited the strongest correlation with PPC (r = 0.83). In summary, both TFs and TIs at 750 and 840 nm showed more pronounced correlations with winter wheat PPC, and most TIs outperformed the original TFs. These results indicate that winter wheat PPC is highly sensitive to red-edge and NIR spectral features, confirming the feasibility of monitoring crop nutrient parameters in these bands.

### 3.3 Winter wheat PPC monitoring using selected spectral features

**Figure 8:**
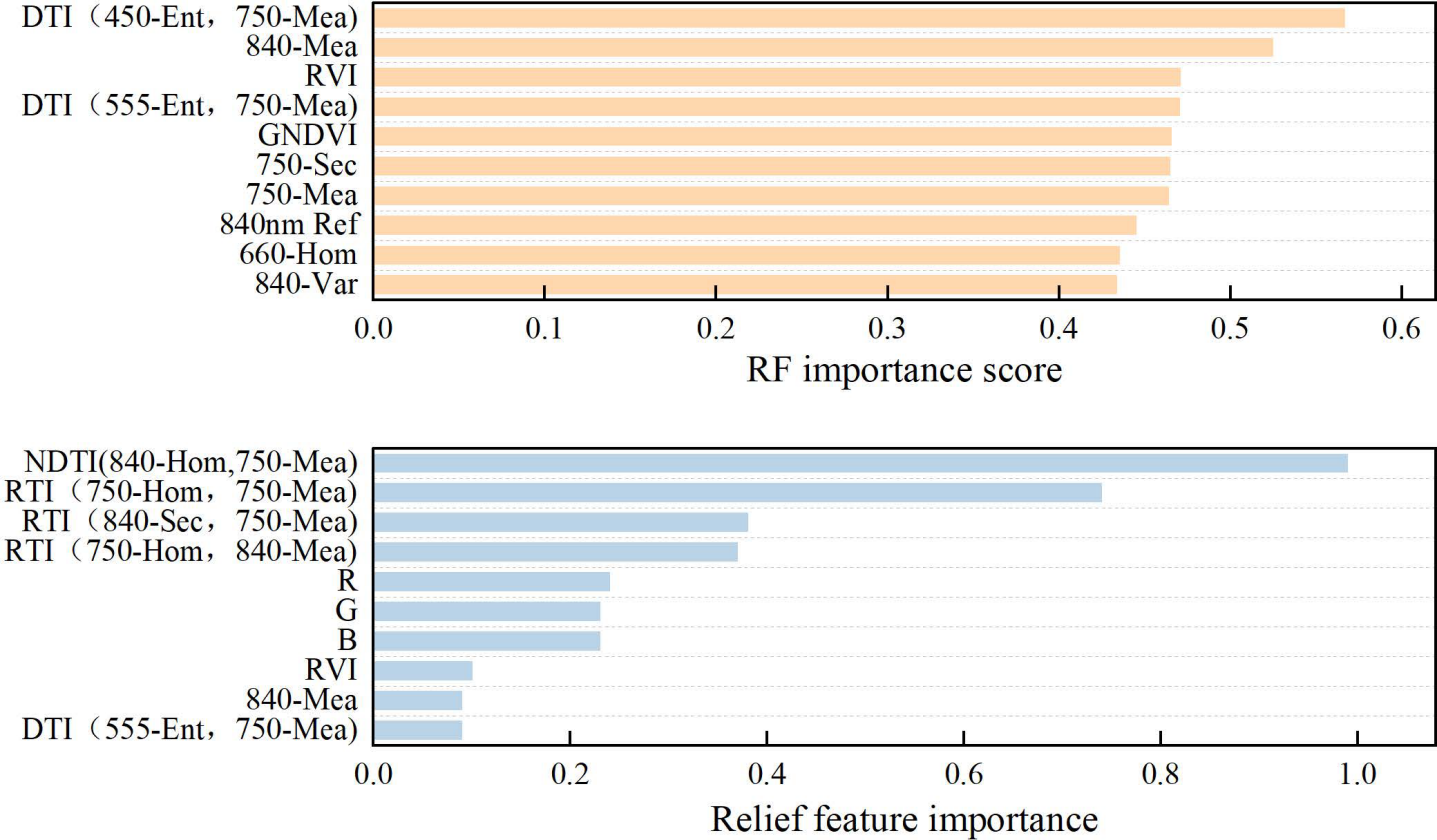
Ranking of importance features of RF and Relief models.

In this study, spectral features were selected using three feature selection methods. RF, SVM, and KNN algorithms were employed to develop winter wheat PPC monitoring models to evaluate the applicability of spectral features for PPC estimation. Pearson correlation analysis was first applied to identify the spectral variables strongly correlated with winter wheat PPC, and different variable combinations were constructed to develop monitoring models (**Table 3**). In terms of RF feature importance ranking, spectral variables were ranked from highest to lowest in importance, with the top 10 features selected for model establishment. For the Relief algorithm, the top 10 spectral features were selected based on descending feature weights, with their corresponding importance scores calculated (**Figure 8**). Based on the features selected by the RF and Relief algorithms, models were further developed using sequentially optimized numbers of input variables (**Table 4**). Validation results highlighted high accuracy with R^2^ >0.8.

**Table 3:** The validation set results of the model for PPC in winter wheat based on correlation screening.

Variable type	Number	RF			SVM			KNN		
		R^2^	RMSE	RPD	R^2^	RMSE	RPD	R^2^	RMSE	RPD
CIs	5	0.26	1.09	1.19	0.05	1.24	1.05	0.19	1.14	1.14
VIs	4	0.58	0.82	1.59	0.65	0.75	1.74	0.79	0.58	2.26
FVC	1	0.60	0.80	1.62	0.02	1.26	1.04	0.21	1.13	1.16
Texture	12	0.63	0.77	1.70	0.69	0.70	1.85	0.74	0.64	2.03
TIs	11	**0.80**	0.56	2.31	**0.82**	0.73	2.33	0.65	0.75	1.73
CIs+VIs	9	0.63	0.77	1.70	0.70	0.70	1.87	0.73	0.66	1.98
CIs+FVC	6	0.67	0.73	1.78	0.49	0.90	1.44	0.25	1.10	1.19
CIs+Texture	17	0.65	0.75	1.73	**0.81**	0.62	2.28	0.67	0.73	1.78
CIs+TIs	16	0.71	0.67	1.93	0.73	0.66	1.99	0.65	0.75	1.74
VIs+FVC	5	0.76	0.62	2.12	0.62	0.79	1.66	0.77	0.61	2.15
VIs+Texture	16	0.70	0.70	1.87	0.72	0.47	1.89	0.73	0.66	1.97
VIs+TIs	15	0.74	0.65	2.00	0.76	0.62	2.09	0.67	0.73	1.79
FVC+Texture	13	0.76	0.62	2.10	0.72	0.67	1.96	0.71	0.68	1.90
FVC+TIs	12	0.77	0.61	2.14	0.72	0.67	1.95	0.69	0.70	1.85
Texture+TIs	23	**0.80**	0.57	2.27	0.74	0.65	1.96	0.69	0.71	1.84
CIs+VIs+FVC	10	**0.81**	0.55	2.37	**0.85**	0.49	2.64	0.75	0.64	2.04
CIs+VIs+Texture	21	0.71	0.69	1.90	0.71	0.68	1.90	0.68	0.72	1.81
CIs+VIs+TIs	20	**0.80**	0.56	2.31	0.75	0.63	2.06	0.66	0.74	1.76
CIs+FVC+Texture	18	0.67	0.73	1.78	**0.84**	0.69	2.51	0.67	0.73	1.78
CIs+FVC+TIs	17	0.72	0.67	1.94	0.70	0.69	1.90	0.67	0.73	1.78
CIs+Texture+TIs	28	0.69	0.71	1.84	0.72	0.57	1.89	0.69	0.70	1.86
VIs+FVC+Texture	17	0.68	0.72	1.80	**0.85**	0.50	2.62	0.72	0.67	1.93
VIs+FVC+TIs	16	**0.81**	0.55	2.36	0.78	0.60	2.17	0.68	0.72	1.81
VIs+Texture+TIs	27	0.70	0.69	1.88	**0.85**	0.36	2.57	0.69	0.70	1.86
FVC+Texture+TIs	24	0.67	0.73	1.79	0.79	0.56	2.18	0.72	0.67	1.93
VIs+FVC+Texture+TIs	28	0.71	0.68	1.92	0.80	0.46	2.22	0.70	0.70	1.86
CIs+FVC+Texture+TIs	29	0.79	0.58	2.24	0.69	0.60	1.80	0.70	0.69	1.88
CIs+VIs+Texture+TIs	32	0.73	0.65	1.99	**0.80**	0.57	2.27	0.70	0.69	1.88
CIs+VIs+FVC+TIs	21	**0.85**	0.50	2.62	0.78	0.60	2.17	0.69	0.71	1.85
CIs+VIs+FVC+Texture	22	0.71	0.68	1.92	0.45	0.90	1.35	0.76	0.63	2.08
All	33	0.76	0.62	2.09	0.76	0.63	2.08	**0.81**	0.55	2.38

Note: Results with a validation set R^2^ greater than 0.8 are presented in bold.

**Table 4:** The results of the model validation set after optimizing variables based on different screening methods.

Screening method	Number	RF			SVM			KNN		
		R^2^	RMSE	RPD	R^2^	RMSE	RPD	R^2^	RMSE	RPD
RF Importance Ranking	10	0.70	0.69	1.89	0.76	0.63	2.08	**0.81**	0.55	2.37
	9	**0.81**	0.56	2.33	0.72	0.51	1.88	**0.87**	0.46	2.85
	8	**0.85**	0.49	2.66	0.68	0.72	1.82	**0.87**	0.47	2.80
	7	0.77	0.60	2.16	**0.89**	0.55	3.00	**0.87**	0.46	2.83
	6	**0.85**	0.50	2.64	**0.91**	0.57	3.37	**0.85**	0.50	2.61
	5	0.72	0.67	1.95	**0.91**	0.37	3.36	**0.86**	0.48	2.70
	4	0.67	0.73	1.79	**0.87**	0.45	2.77	**0.85**	0.48	2.69
	3	0.67	0.73	1.78	**0.92**	0.36	3.48	**0.85**	0.49	2.67
Relief	10	0.71	0.68	1.92	0.73	0.43	1.98	0.76	0.62	2.11
	9	0.75	0.64	2.04	**0.84**	0.43	2.51	**0.83**	0.44	2.45
	8	0.68	0.72	1.81	0.66	0.49	1.77	0.67	0.73	1.78
	7	0.76	0.62	2.12	0.36	0.67	1.29	0.62	0.78	1.66
	6	0.76	0.63	2.08	0.75	0.42	2.04	0.60	0.80	1.62
	5	0.61	0.79	1.65	0.43	0.63	1.36	0.54	0.86	1.51
	4	0.59	0.81	1.61	0.58	0.71	1.55	0.53	0.87	1.49
	3	0.41	0.98	1.33	0.26	0.72	1.20	0.46	0.93	1.40

Results with a validation set R^2^ greater than 0.8 are presented in bold.

For models constructed using Pearson correlation-selected features, RF achieved the highest accuracy when combining CIs, VIs, FVC, and TIs (R^2^ = 0.85, RMSE = 0.50, RPD = 2.62). The R^2^ increases of 226.9%, 46.6%, 41.7%, and 6.3% compared with single-variable models. Models based on CIs + VIs + FVC and VIs + FVC + TIs demonstrated comparable performance (both R^2^ = 0.81). For SVM, the optimal performance was achieved using CIs + VIs + FVC, VIs + FVC + TFs, and VIs + TFs + TIs (all R^2^ = 0.85), with varying degrees of improvement relative to single-variable models. The CIs + FVC + TFs combination also exhibited a strong predictive ability (R^2^ = 0.84, RMSE = 0.69, RPD = 2.51). KNN achieved the best performance when all variables were included (R^2^ = 0.81, RMSE = 0.55, RPD = 2.38). For RF importance-selected models, RF reached peak accuracy with six and eight input variables (both R^2^ = 0.85), followed by nine variables (R^2^ = 0.81). SVM exhibited stable performance with three to seven variables and achieved optimal results with three variables (R^2^ = 0.92, RMSE = 0.36, RPD = 3.48). The KNN models with three to ten variables all achieved R^2^ values exceeding 0.8, with the highest accuracy observed for models with seven to nine variables (all R^2^ = 0.87). In contrast, for Relief-selected models, all RF models yielded R^2^ values below 0.8. The SVM and KNN achieved their optimal performance with nine variables (R^2^ = 0.84, RMSE = 0.43, RPD = 2.51; KNN: R^2^ = 0.83, RMSE = 0.44, RPD = 2.45). Overall, variable optimization under different feature selection strategies effectively improved winter wheat PPC estimation accuracy, with Pearson correlation and RF importance yielding superior performance. These results further verify that multi-feature fusion effectively boosts the predictive performance of PPC estimation models.

**Figure 9:**
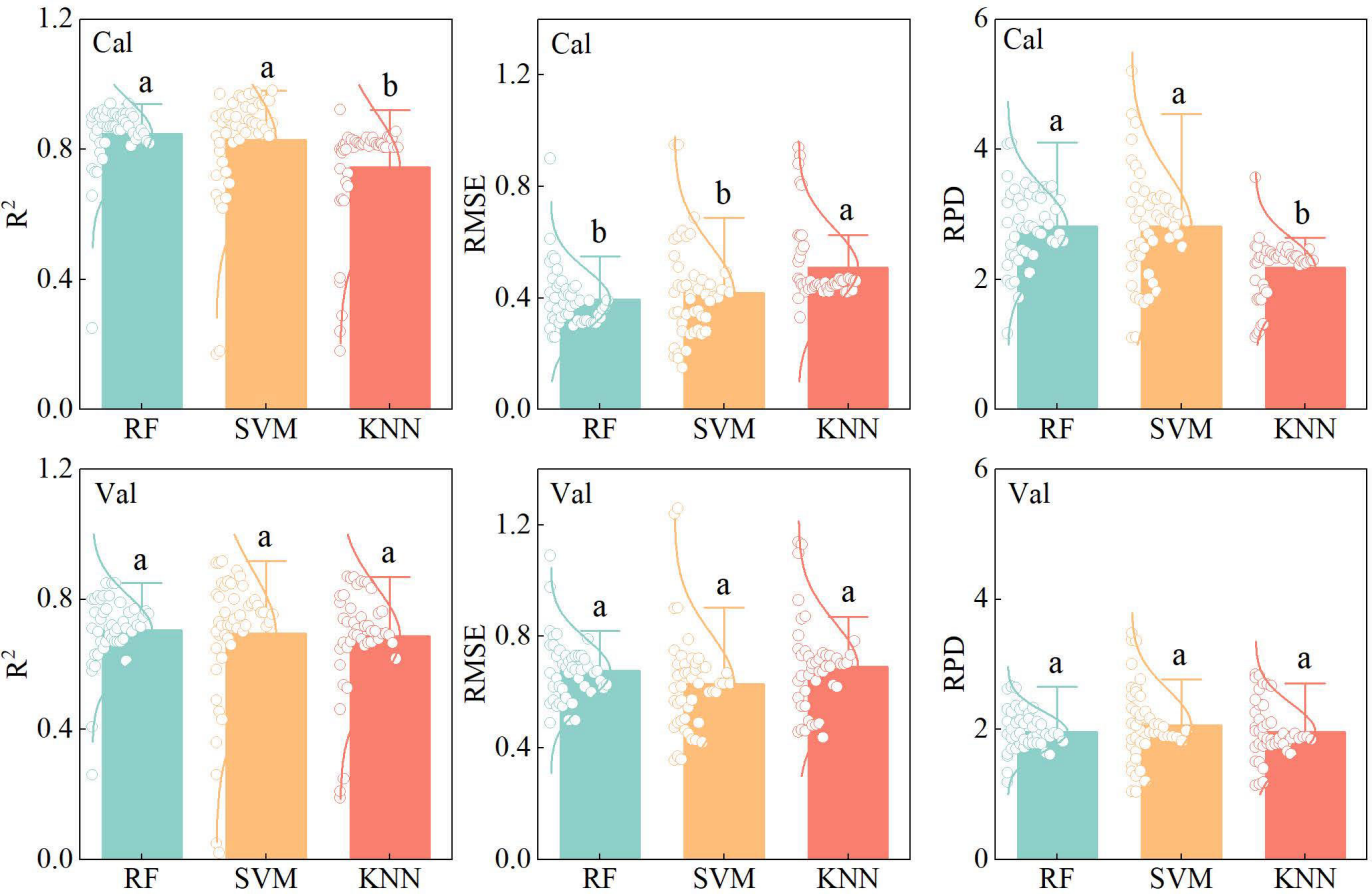
Comparison of the calibration sets and validation sets results of models constructed based on different machine learning algorithms. Between-model differences were assessed via one-way ANOVA and Duncan’ s multiple range test (p< 0.05). Different lowercase letters indicate significant differences among treatments at the same growth stage.

**Figure 10:**
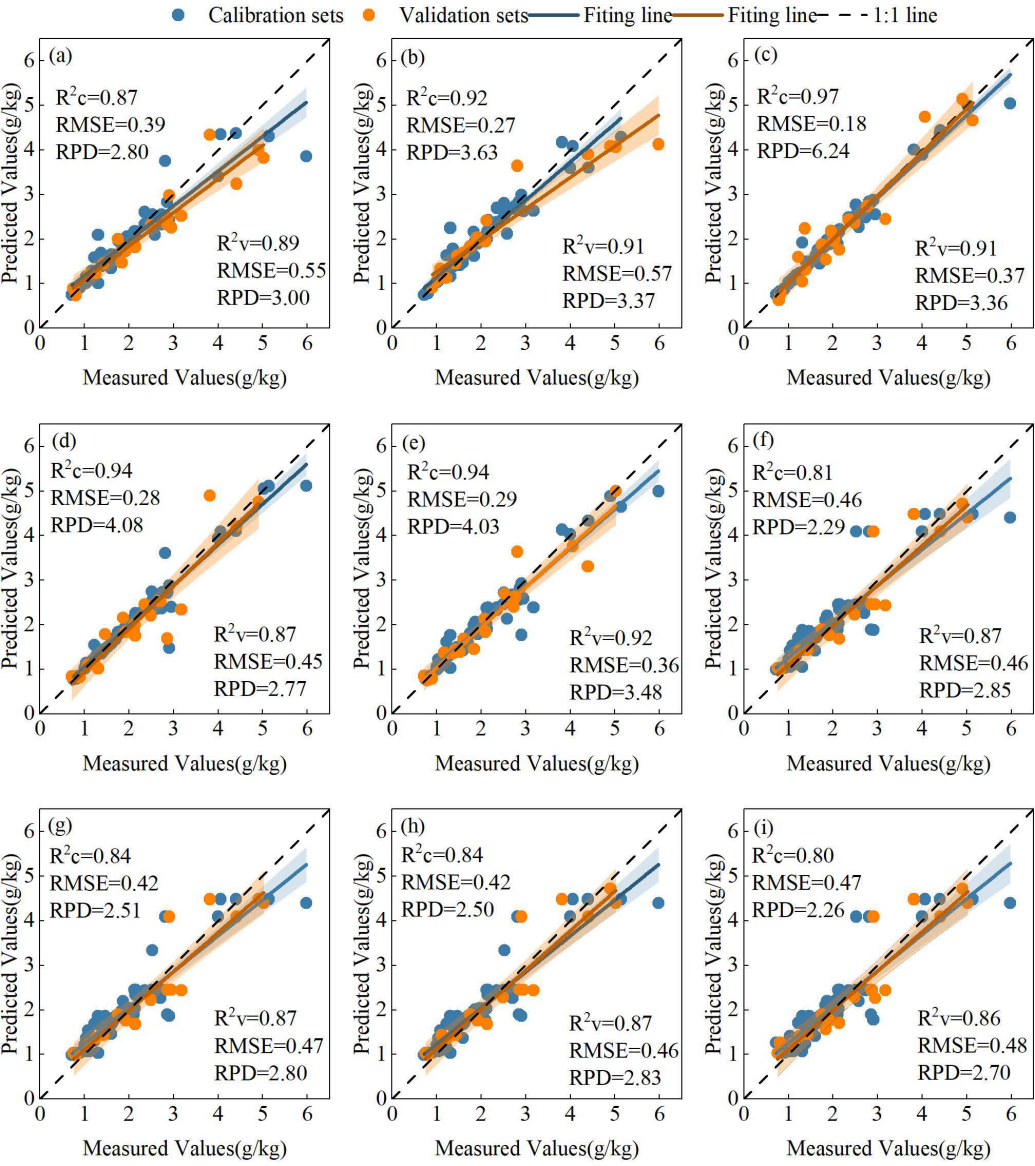
Scatter plots of the optimal models for PPC in winter wheat. All nine models were developed using variables selected based on RF importance ranking. **(a-e)** SVM models, featuring 7, 6, 5, 4,and 3 input variables respectively; **(f-i)** KNN models, featuring 9, 8, 7, and 5 input variables respectively.

**Figure 11:**
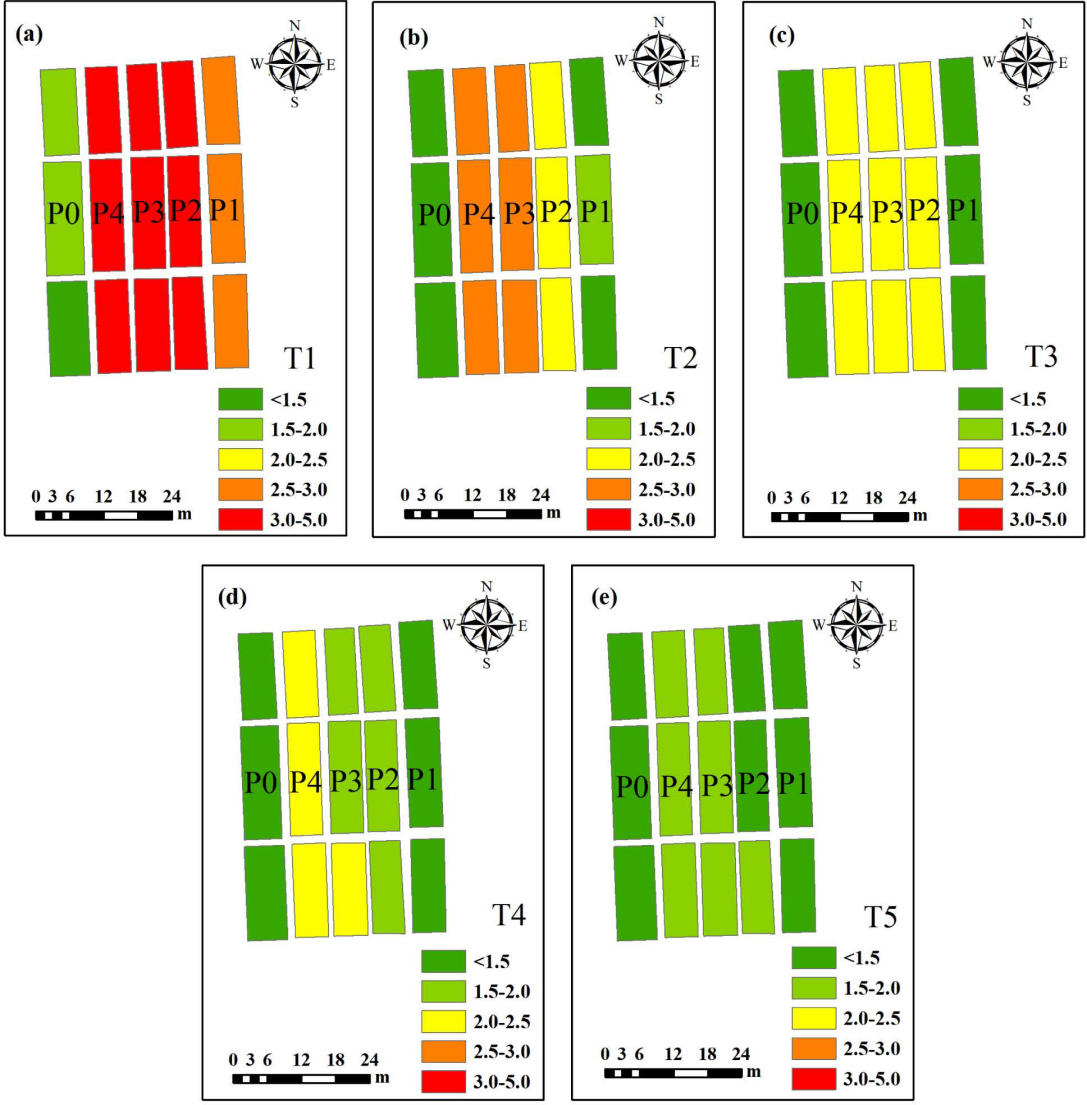
Inversion diagrams of PPC in winter wheat at different growth stages based on the optimal model. **(a–e)** respectively represent the jointing stage, the booting stage, the flowering stage, the grain filling stage and the maturity stage.

From the perspective of different machine learning algorithms, the R^2^, RMSE, and RPD values of calibration and validation sets were compared across models (**Figure 9**). In the calibration set, RF achieved the highest mean R^2^ value (0.85), followed by SVM, whereas KNN exhibited the lowest mean R^2^ (0.74). A similar pattern was observed in the validation set, where RF demonstrated the strongest fitting and generalization performance, indicating its overall superiority compared with SVM and KNN. To further illustrate the model fitting effectiveness, nine optimal estimation models were selected, all of which exhibited validation R^2^ values exceeding 0.85. The scatter plots of calibration and validation results for these models were compared with 1:1 reference lines (**Figure 10**). Notably, the SVM model based on RF importance-selected variables achieved the best overall performance with three input variables (R^2^c=0.94, RMSEc=0.29, RPDc=4.03; R^2^v=0.92, RMSEv=0.36, RPDv=3.48), including DTI (450-Ent, 750-Mea), 840-Mea, and RVI (**Figure 10e**). On the basis of the best-performing model, spatial distribution maps illustrating winter wheat PPC were constructed across the jointing, booting, heading, filling, and maturity periods (**Figure 11**). The results indicated that PPC gradually decreased with advancing growth stages, and differences among P application treatments were more pronounced during the early growth stages.

## 4 Discussion

### 4.1 Response relationships between spectral characteristic parameters and PPC under different P application treatments

To elucidate the impacts of different P application treatments on winter wheat PPC and spectral characteristics, variations in both parameters across treatments and growth stages were analyzed. The results indicated that the differences in PPC between P-applied and non-P treatments were more pronounced during early growth stages. This directly reflects the essential role of P in crop growth and development, with the early growth stage representing a critical period for root expansion and nutrient accumulation (Liu, 2021). The present study was conducted on a long-term stationary P management platform. After 17 years of differential P fertilizer inputs, substantial differences in soil P supply capacity developed, which directly influenced crop P uptake. This finding aligns with the report by Zhou et al. (2024), who demonstrated that non-P treatments resulted in insufficient soil available P supply, whereas P application treatments could meet plant P demand during early growth stages, leading to higher P concentrations. As growth progressed, PPC differences among treatments gradually declined, along with a general reduction in overall PPC, which is broadly consistent with previous findings (Yan et al., 2023). Winter wheat biomass generally approaches its maximum in late growth stages, and plant dry weight under different P treatments tends to be relatively comparable. This may trigger a biomass dilution effect and potentially weaken the disparities in PPC (Xue et al., 2021). Abbas et al. (2018) confirmed that the decreased P concentration in mature plants is attributed to P remobilization from vegetative to reproductive organs, which may partly lower the overall PPC level.

Multispectral reflectance and most TFs differed markedly under P application in red-edge and NIR bands, indicating that P regulates vegetation spectral signatures across these key spectral ranges. Consistent with Xie et al. (2024), P is essential for chlorophyll synthesis, and P fertilization significantly enhances leaf chlorophyll content in winter wheat. As the red-edge band represents the transition between chlorophyll absorption and reflectance (Yao et al., 2025), it may be particularly sensitive to P-induced variations. P deficiency induces leaf water loss, cell structure shrinkage, leaf chlorophyll degradation, and anthocyanin accumulation (Li et al., 2022). These physiological alterations likely increase visible reflectance, decrease NIR reflectance, and cause a blue shift of the red edge (Zhang et al., 2024b). Meanwhile, P limitation may restrict tillering and reduce population density and canopy coverage. Such variations could aggravate soil background interference and weaken the identification of chlorophyll-related spectral features (Shu et al., 2023). Moreover, P-deficiency-induced canopy structural disorder may lower NIR scattering (Zhang et al., 2019). The results showed that NIR reflectance was higher with P fertilization relative to the control. This difference may be explained by variations in leaf water content, cell structure and canopy architecture, which collectively modulate NIR spectral characteristics (Deng et al., 2019; Xing et al., 2008). However, no significant variations in spectral TFs were found among different P application rates. This may stem from the fact that TFs characterize crop morphology, yet internal nutrient differences are hardly detected by texture alone (Sankaran et al., 2015). Accordingly, future studies could adopt higher-spatial-resolution imagery to further explore P effects on crop morphological traits. In this experiment, gradient P treatments were established with constant nitrogen application. Adequate and consistent nitrogen supply minimized inter-treatment differences in plant nitrogen status and maintained stable spectral performance. Nitrogen directly affects visible light absorption and reflectance by regulating chlorophyll content (Mu and Chen, 2021). As a core component of ATP and ADP (Khan et al., 2023), P fundamentally regulates crop growth and canopy properties, thereby modifying spectral characteristics (Nguyen et al., 2022; Chen et al., 2019). This experimental design effectively eliminated the indirect effects of nitrogen on P-related spectral responses and prevented nitrogen from acting as a confounding factor.

Correlation analysis further indicated that GRI, VARI, MGRVI, TGI, NDRE, and CIred edge were strongly correlated with winter wheat PPC at the maturity stage. This strong correlation may be attributed to the peak regulatory effect of P on leaf biochemical components and cell structures at this stage, as these indices were highly sensitive to P-induced changes in leaf spectral traits (Xu et al., 2022a). The maturity stage is pivotal for photosynthate translocation and grain filling in winter wheat. P deficiency impairs photosynthate transport, accelerates chlorophyll degradation, and induces anthocyanin accumulation (Chen et al., 2024; Li et al., 2022). GRI, VARI, MGRVI, and TGI are derived from green and red band reflectance, thus directly capturing changes in leaf chlorophyll and anthocyanin contents (Ma et al., 2024). In contrast, NDRE and CIred edge are red-edge indices; chlorophyll degradation caused by P deficiency at maturity triggers a blue shift and slope reduction of the red-edge position (Li et al., 2024). Notably, the physiological traits specific to the maturity stage further strengthened the vegetation index-P content correlation. This is likely because substantial P is remobilized from vegetative organs to grains during this period, pushing plant P supply-demand imbalance to a maximum (Xu et al., 2022a). In addition, both TFs and TIs exhibited stronger correlations with PPC in the red edge and NIR bands, with most TIs outperforming the original TFs in terms of correlation strength. This finding was consistent with the observed spectral response patterns for P concentration and further confirmed the sensitivity of the red edge and NIR spectral regions to PPC variations. Li et al. (2019) reported that TIs constructed through combined operations on original TFs could reduce the redundant information and enhance the sensitivity to canopy texture differences. Therefore, the construction of TIs was considered effective for optimizing spectral-texture information and improving the detection of PPC-related canopy variations.

### 4.2 Potential of feature selection and fusion for winter wheat PPC monitoring

This study employed Pearson correlation analysis, RF importance ranking, and the Relief algorithm for spectral feature selection to examine their effects on the PPC estimation of winter wheat. Correlation-based selection indicated that combining different types of spectral features improved the model estimation accuracy, with optimal performance achieved by fusing FVC, TFs, and TIs. Numerous prior studies have illustrated that integrating diverse spectral features can help mitigate the inherent limitations of single variables and capture complementary information related to crop growth (Zhang et al., 2025). FVC reflects the canopy structure and growth status, which are inherently associated with P concentration (Qiao et al., 2024). TFs characterize the spatial distribution of canopy imagery, whereas TIs further enhance the relevance of TFs to PPC by reducing noise interference and improving sensitivity (Yun et al., 2024). Consequently, feature fusion enables the construction of multi-dimensional spectral information encompassing growth status, canopy structure, and resistance to external interference, thereby improving estimation accuracy. For RF importance-based feature selection, the model constructed using DTI (450-Ent, 750-Mea), 840-Mea, and RVI achieved the best performance (R^2^v=0.92, RMSEv=0.36, RPDv=3.48). This superior performance may be attributed to DTI (450-Ent, 750-Mea). The 450 nm blue band may be sensitive to variations in leaf pigments under P stress (Zhao et al., 2024). Its entropy texture effectively characterizes canopy spectral heterogeneity and spatial disorder (Sarsen et al., 2026), which may indicate nutrient-deficient growth traits of winter wheat. The 750 nm red-edge band is closely associated with crop photosynthesis and nutrient metabolism (Yu et al., 2019). Its mean texture may reflect subtle canopy structural variations caused by P imbalance (Jin et al., 2026). As a coupled feature, DTI integrates pigment-related spectral signals and canopy structural information simultaneously. It may make up for the insufficient representation of single spectral or texture indicators under P stress (Xu et al., 2022b; Zhu et al., 2023). Both 840-Mea and RVI are derived from the NIR band. NIR signals are likely correlated with crop growth and biomass accumulation, and such vegetation traits are closely regulated by plant P supply (Li et al., 2016). These results demonstrate that appropriate feature selection and fusion can substantially enhance PPC estimation accuracy. In addition, the Relief algorithm-based model exhibited relatively good performance when nine spectral variables were selected, suggesting that most spectral features were closely correlated with PPC. However, its overall accuracy was lower than that of the other two methods. Previous studies note that spectral datasets suffer from high redundancy due to correlated features. The Relief algorithm performs poorly with high-dimensional redundant data, degrading model generalization (Thorp et al., 2017; Cheng et al., 2023). In contrast, Pearson correlation analysis directly selects spectral features strongly correlated with P concentration. RF importance ranking automatically reduces redundancy via ensemble decision trees and improves selection accuracy by accounting for feature interactions (Ban et al., 2021; Li et al., 2018; Zhou et al., 2023). Accordingly, future studies should consider applying dimensionality reduction to spectral data prior to Relief-based feature selection to alleviate redundancy and improve model robustness. Notably, this study employed three feature selection methods to screen sensitive features for model input and the models achieved high accuracy under the current experimental condition of this study. However, the field experiment was limited to a single site, one growing season, and a single wheat cultivar. Accordingly, the spatial and temporal generalization of the established models requires further validation. Future work will conduct multi-site, multi-year and multi-cultivar field trials. Further optimization of feature combinations and modeling strategies will effectively improve the robustness and universality of UAV-based crop P monitoring approaches.

### 4.3 Effects of model algorithms on winter wheat PPC monitoring

In this study, winter wheat PPC monitoring models were developed using RF, SVM, and KNN algorithms to evaluate their suitability for monitoring P concentrations. A comparative analysis of model performance demonstrated that RF outperformed SVM and KNN overall, confirming its advantage in modeling high-dimensional spectral data. The ensemble learning of RF captures nonlinear correlations between spectral variables and PPC, mitigates data redundancy, and maintains stable performance in crop physiological parameter estimation (Meng et al., 2025). Notably, the optimal PPC estimation model was an SVM model constructed using DTI (450-Ent, 750-Mea), 840-Mea, and RVI, which achieved the highest predictive accuracy (R^2^c=0.94, RMSEc=0.29, RPDc=4.03; R^2^v=0.92, RMSEv=0.36, RPDv=3.48). This result indicates the strong explanatory capability of SVM for variations in winter wheat PPC. Similar findings were reported by Teshome et al. (2023), who emphasized the robustness of SVM in handling high-dimensional datasets. Moreover, SVM performs well with small datasets and models nonlinear relationships through structural risk minimization, achieving stable predictions (Yuan and Tan, 2010). Accordingly, the superior performance of the SVM model in this study can be attributed to the limited sample size and pronounced nonlinear relationships between spectral features and PPC. In contrast, KNN exhibited the lowest overall performance among the three algorithms. Existing studies indicate spectral datasets involve many correlated bands. KNN’ s distance metric performs poorly in high-dimensional spaces, lowering prediction accuracy (Chen et al., 2022). Moreover, the simple structure of KNN renders it more sensitive to noise and outliers, whose neighborhood effects may further amplify prediction bias. Thus, future research may enhance KNN performance by optimizing neighbor selection and adopting dimensionality reduction.

## 5 Conclusions

To evaluate the feasibility of monitoring winter wheat PPC through the fusion of multi-source UAV image features, this study extracted CIs, FVC, VIs, TFs, and TIs from UAV RGB and multispectral images. Sensitive spectral features were screened using Pearson correlation analysis, RF importance ranking, and the Relief algorithm, with RF, SVM, and KNN employed to build winter wheat PPC monitoring models. Effective monitoring of winter wheat PPC was achieved in the study area.

Correlation analysis demonstrated that GRI, VARI, MGRVI, TGI, NDRE, and CIred edge exhibited strong correlations with winter wheat PPC at the maturity stage. Both TFs and TIs showed stronger correlations with PPC in the red edge and NIR bands, confirming the feasibility of UAV-based spectral monitoring of PPC. Among feature selection methods, RF importance ranking and Pearson correlation analysis yielded higher model estimation accuracy. Fusion-based spectral feature selection effectively improved winter wheat PPC estimation precision. Of all models, the SVM model based on RF importance-selected variables exhibited the optimal performance (R^2^c=0.94, RMSEc=0.29, RPDc=4.03; R^2^v=0.92, RMSEv=0.36, RPDv=3.48), with the input variables including DTI (450-Ent, 750-Mea), 840-Mea, and RVI. These results demonstrate that variable optimization via RF importance screening enhanced winter wheat PPC estimation accuracy and highlighted the application potential of SVM for PPC prediction. Overall, this study provides data-driven insights for UAV-based monitoring of plant P nutritional status and their spectral responses under local experimental conditions.

## Data Availability

The datasets presented in this article are not readily available because the data are currently confidential and cannot be made publicly available. Requests to access the datasets should be directed to YS, susu121hbxt@163.com.

## References

[B1] Al-Abbas A. Barr R. Hall J. Crane F. Baumgardner M. (1974). Spectra of normal and nutrient-deficient maize leaves. Agron. 66, 16–20. doi:10.2134/agronj1974.00021962006600010005x

[B2] Ban S. Tian M. Chang Q. Wang Q. Li F. (2021). Estimation of rice leaf phosphorus content using UAV-based hyperspectral images. Trans. Chin. Soc Agric. Mach. 52, 163–171. doi:10.6041/j.issn.1000-1298.2021.08.016

[B3] Bendig J. Yu K. Aasen H. Bolten A. Bennertz S. Broscheit J. . (2015). Combining UAV-based plant height from crop surface models, visible, and near infrared vegetation indices for biomass monitoring in barley. Int. J. Appl. Earth Obs. Geoinf. 39, 79–87. doi:10.1016/j.jag.2015.02.012. PMID: 38826717

[B4] Cao Y. Lin M. Guo Z. Xiao W. Ma D. Xu T. (2021). Unsupervised GMM for rice segmentation with UAV images based on Lab color space. Trans. Chin. Soc Agric. Mach. 52, 162–169. doi:10.6041/j.issn.1000-1298.2021.01.018

[B5] Carlson T. Ripley D. (1997). On the relation between NDVI, fractional vegetation cover, and leaf area index. Remote Sens. Environ. 62, 241–252. doi:10.1016/S0034-4257(97)00104-1

[B6] Chen Z. Jia K. Li Q. Xiao C. Wei D. Zhao X. . (2022). Hybrid feature selection for cropland identification using GF-5 satellite image. Natl. Remote Sens. Bull. 26, 1383–1394. doi:10.11834/jrs.20220458

[B7] Chen W. Yang G. Meng Y. Feng H. Li H. Tang A. . (2024). Estimation of winter wheat stem biomass by a novel two-component and two-parameter stratified model using proximal remote sensing and phenological variables. Remote Sens. 16, 4300. doi:10.3390/rs16224300. PMID: 30654563

[B8] Chen X. Zhang W. Liang X. Liu Y. Xu S. Zhao Q. . (2019). Physiological and developmental traits associated with the grain yield of winter wheat as affected by phosphorus fertilizer management. Sci. Rep. 9, 16580. doi:10.1038/s41598-019-53000-z. PMID: 31719561 PMC6851383

[B9] Cheng F. Wang W. Zhang Z. (2023). Hierarchical subspace ReliefF feature selection algorithm for high-dimensional small sample data. J. Nanjing Univ. (Nat. Sci.) 59, 928–936.

[B10] Cordell D. Drangert J. White S. (2009). The story of phosphorus: global food security and food for thought. Glob. Environ. Change: Hum. Policy Dimens. 19, 292–305. doi:10.1016/j.gloenvcha.2008.10.009. PMID: 38826717

[B11] Dash J. Curran P. (2004). The MERIS terrestrial chlorophyll index. Int. J. Remote Sens. 25, 5403–5413. doi:10.1080/0143116042000274015. PMID: 37339054

[B12] Deng L. Zhang F. Zhang H. Zhang X. Yuan J. (2019). Analysis of the spectral characteristics of Haloxylon Ammodendron under water stress. Spectrosc. Spectr. Anal. 39, 210–215. doi:10.3964/j.issn.1000-0593(2019)01-0210-06. PMID: 42102434

[B13] Feng F. Fan Y. Yue J. Bian M. Liu Y. Chen R. . (2025). Estimation of potato above-ground biomass based on the VGC-AGB model and deep learning. Comput. Electron. Agric. 232, 110122. doi:10.1016/j.compag.2025.110122. PMID: 38826717

[B14] Feng J. Pan Y. Xiong Y. Wu C. Xiao D. (2024). Rice key growth stage identification based on mRMR-XGBoost. Trans. Chin. Soc Agric. Eng. (Trans. CSAE) 40, 111–118. doi:10.11975/j.issn.1002-6819.202312001

[B15] Fu G. Li C. Liu W. Pan K. He J. Li W. (2025). Maize yield estimation based on UAV multispectral monitoring of canopy LAI and WOFOST data assimilation. Eur. J. Agron. 168, 127614. doi:10.1016/j.eja.2025.127614. PMID: 38826717

[B16] Gamon J. Surfus J. (1999). Assessing leaf pigment content and activity with a reflectometer. New Phytol. 143, 105–117. doi:10.1046/j.1469-8137.1999.00424.x. PMID: 37945311

[B17] Gan Y. Wang Q. Matsuzawa T. Song G. Iio A. (2023). Multivariate regressions coupling colorimetric and textural features derived from UAV-based RGB images can trace spatiotemporal variations of LAI well in a deciduous forest. Int. J. Remote Sens. 44, 4559–4577. doi:10.1080/01431161.2023.2208709. PMID: 37339054

[B18] Gitelson A. Merzlyak M. Lichtenthaler H. (1996). Detection of red edge position and chlorophyll content by reflectance measurements near 700 nm. J. Plant Physiol. 148, 501–508. doi:10.1016/S0176-1617(96)80285-9

[B19] Guo P. Shi Z. Li M. Luo W. Cha Z. (2018). A robust method to estimate foliar phosphorus of rubber trees with hyperspectral reflectance. Ind. Crops Prod. 126, 1–12. doi:10.1016/j.indcrop.2018.09.055. PMID: 38826717

[B20] Hang Y. Su H. Yu Z. Liu H. Guan H. Kong F. (2021). Estimation of rice leaf area index combining UAV spectrum, texture features and vegetation coverage. Trans. Chin. Soc Agric. Eng. (Trans. CSAE) 37, 64–71. doi:10.11975/j.issn.1002-6819.2021.09.008

[B21] Hastie T. Tibshirani R. Friedman J. (2009). The elements of statistical learning: data mining, inference, and prediction. Springer. doi:10.1007/978-0-387-84858-7. PMID: 30311153

[B22] Huang N. Lun F. Chen X. Gao R. Bi P. Men J. . (2025). The synergistic effects of climate change and fertilizer on crop yield: evidence from winter wheat in China. J. Sci. Food Agric. 105, 8787–8797. doi:10.1002/jsfa.70124. PMID: 40827442

[B23] Hunt E. Doraiswamy P. McMurtrey J. Daughtry C. Perry E. Akhmedov B. (2013). A visible band index for remote sensing leaf chlorophyll content at the canopy scale. Int. J. Appl. Earth Obs. Geoinf. 21, 103–112. doi:10.1016/j.jag.2012.07.020. PMID: 38826717

[B24] Jiang J. Ji H. Zhou G. Pan R. Zhao L. Duan Z. . (2025). Non-destructive monitoring of tea plant growth through UAV spectral imagery and meteorological data using machine learning and parameter optimization algorithms. Comput. Electron. Agric. 229, 109795. doi:10.1016/j.compag.2024.109795. PMID: 38826717

[B25] Jiao C. Xie X. Hao C. Chen L. Xie Y. Garg V. . (2024). Pan-genome bridges wheat structural variations with habitat and breeding. Nature 637, 1–10. doi:10.1038/s41586-024-08277-0. PMID: 39604736

[B26] Jin J. Cheng X. Cai Y. Qin Y. Zhu Q. Wang W. . (2026). Guiding VI selection for phenology monitoring: differential sensitivity of vegetation indices to temporal dynamics in canopy leaf area and pigment. Remote Sens. Environ. 335, 115296. doi:10.1016/j.rse.2026.115296. PMID: 38826717

[B27] Khan F. Siddique A. Shabala S. Zhou M. Zhao C. (2023). Phosphorus plays key roles in regulating plants’ physiological responses to abiotic stresses. Plants 12, 2861. doi:10.3390/plants12152861. PMID: 37571014 PMC10421280

[B28] Kira K. Rendell L. (1992). A practical approach to feature selection. In Proc. 9th Natl. Conf. Artif. Intell., 249–254. doi:10.1016/B978-1-55860-247-2.50037-1. PMID: 38826717

[B29] Li D. Chen J. Yu W. Zheng H. Yao X. Zhu Y. . (2024). A chlorophyll-constrained semi-empirical model for estimating leaf area index using a red-edge vegetation index. Comput. Electron. Agric. 220, 108891. doi:10.1016/j.compag.2024.108891. PMID: 38826717

[B30] Li Z. Cheng Q. Chen L. Yang J. Zhai W. Mao B. . (2025b). Enhancing winter wheat plant nitrogen content prediction across different regions: integration of UAV spectral data and transfer learning strategies. Comput. Electron. Agric. 234, 110322. doi:10.1016/j.compag.2025.110322. PMID: 38826717

[B31] Li J. Li J. Zhao D. Cao Q. Yu F. Cao S. . (2025a). High-throughput method for improving rice AGB estimation based on UAV multi-source remote sensing image feature fusion and ensemble learning. Front. Plant Sci. 16, 1576212. doi:10.3389/fpls.2025.1576212. PMID: 40303858 PMC12038451

[B32] Li D. Wang C. Jiang H. Peng Z. Yang J. Su Y. . (2018). Monitoring litchi canopy foliar phosphorus content using hyperspectral data. Comput. Electron. Agric. 154, 176–186. doi:10.1016/j.compag.2018.09.007. PMID: 38826717

[B33] Li L. Wang S. Ren T. Ma Y. Wei Q. Gao W. . (2016). Evaluating models of leaf phosphorus content of winter oilseed rape based on hyperspectral data. Trans. Chin. Soc Agric. Eng. (Trans. CSAE) 32, 209–218. doi:10.11975/j.issn.1002-6819.2016.14.028

[B34] Li P. Yu J. Feng N. Weng J. Rehman A. Huang J. . (2022). Physiological and transcriptomic analyses uncover the reason for the inhibition of photosynthesis by phosphate deficiency in Cucumis melo L. Int. J. Mol. Sci. 23, 12073. doi:10.3390/ijms232012073. PMID: 36292929 PMC9603772

[B35] Li S. Yuan F. Ata-UI-Karim S. Zheng H. Cheng T. Liu X. . (2019). Combining color indices and textures of UAV-based digital imagery for rice LAI estimation. Remote Sens. 11, 1763. doi:10.3390/rs11151763. PMID: 30654563

[B36] Liang D. Guan Q. Huang W. Huang L. Yang G. (2013). Remote sensing inversion of leaf area index based on support vector machine regression in winter wheat. Trans. Chin. Soc Agric. Eng. 29, 117–123. doi:10.3969/j.issn.1002-6819.2013.07.015

[B37] Liu D. (2021). Root developmental responses to phosphorus nutrition. J. Integr. Plant Biol. 63, 1065–1090. doi:10.1111/jipb.13090. PMID: 33710755

[B38] Liu J. Zhu Y. Song L. Su X. Li J. Zheng J. . (2023). Optimizing window size and directional parameters of GLCM texture features for estimating rice AGB based on UAVs multispectral imagery. Front. Plant Sci. 14, 1284235. doi:10.3389/fpls.2023.1284235. PMID: 38192693 PMC10773816

[B39] Ma G. Satheesh V. Lei M. (2022). Intracellular phosphate sensing in plants. Mol. Plant 15, 1831–1833. doi:10.1016/j.molp.2022.11.002. PMID: 36348624

[B40] Ma R. Zhang N. Zhang X. Bai T. Yuan X. Bao H. . (2024). Cotton Verticillium wilt monitoring based on UAV multispectral-visible multi-source feature fusion. Comput. Electron. Agric. 217, 108628. doi:10.1016/j.compag.2024.108628. PMID: 38826717

[B41] Mali S. Scobie M. Baillie J. Plant C. Shammi S. Das A. (2025). Integrating UAV-based multispectral and thermal infrared imageries with machine learning for predicting water stress in winter wheat. Prec. Agric. 26, 44. doi:10.1007/s11119-025-10239-z. PMID: 30311153

[B42] Meng L. Ming B. Liu Y. Nie C. Fang L. Zhou L. . (2025). Maize biomass estimation by integrating spectral, structural, and textural features from unmanned aerial vehicle data. Eur. J. Agron. 168, 127647. doi:10.1016/j.eja.2025.127647. PMID: 38826717

[B43] Milton N. Eiswerth B. Ager C. (1991). Effect of phosphorus deficiency on spectral reflectance and morphology of soybean plants. Remote Sens. Environ. 36, 121–127. doi:10.1016/0034-4257(91)90034-4

[B44] Motohka T. Nasahara K. Oguma H. Tsuchida S. (2010). Applicability of green-red vegetation index for remote sensing of vegetation phenology. Remote Sens. 2, 2369–2387. doi:10.3390/rs2102369. PMID: 30654563

[B45] Mu X. Chen Y. (2021). The physiological response of photosynthesis to nitrogen deficiency. Plant Physiol. Biochem. 158, 76–82. doi:10.1016/j.plaphy.2020.11.019. PMID: 33296848

[B46] Naito H. Ogawa S. Valencia M. Mohri H. Urano Y. Hosoi F. . (2017). Estimating rice yield related traits and quantitative trait loci analysis under different nitrogen treatments using a simple tower-based field phenotyping system with modified single-lens reflex cameras. ISPRS J. Photogramm. Remote Sens. 125, 50–62. doi:10.1016/j.isprsjprs.2017.01.010. PMID: 38826717

[B47] Nguyen V. Palmer L. Stangoulis J. (2022). Higher photochemical quenching and better maintenance of carbon dioxide fixation are key traits for phosphorus use efficiency in the wheat breeding line, RAC875. Front. Plant Sci. 12, 816211. doi:10.3389/fpls.2021.816211. PMID: 35185965 PMC8854500

[B48] Niu Y. Song X. Zhang L. Xu L. Wang A. Zhu Q. (2025). Enhancing model accuracy of UAV-based biomass estimation by evaluating effects of image resolution and texture feature extraction strategy. IEEE J. Sel. Top. Appl. Earth Obs. Remote Sens. 18, 878–891. doi:10.1109/JSTARS.2024.3501673. PMID: 25079929

[B49] Oliveira H. Castro L. Sousa C. Alves Júnior L. Mesquita M. Silva J. . (2024). Geotechnologies in biophysical analysis through the applicability of the UAV and Sentinel-2A/MSI in irrigated area of common beans: accuracy and spatial dynamics. Remote Sens. 16, 1254. doi:10.3390/rs16071254. PMID: 30654563

[B50] Prabhakara K. Hively W. Mccarty G. (2015). Evaluating the relationship between biomass, percent groundcover and remote sensing indices across six winter cover crop fields in Maryland, United States. Int. J. Appl. Earth Obs. Geoinf. 39, 88–102. doi:10.1016/j.jag.2015.03.002. PMID: 38826717

[B51] Prado L. Marques Ramos A. Roberto Pereira D. Saito Moriya É. Nobuhiro Imai N. Takashi Matsubara E. . (2019). Predicting canopy nitrogen content in citrus-trees using random forest algorithm associated to spectral vegetation indices from UAV-imagery. Remote Sens. 11, 2925. doi:10.3390/rs11242925. PMID: 30654563

[B52] Qiao L. Gao D. Zhang J. Li M. Sun H. Ma J. (2020). Dynamic influence elimination and chlorophyll content diagnosis of maize using UAV spectral imagery. Remote Sens. 12, 2650. doi:10.3390/rs12162650. PMID: 30654563

[B53] Qiao D. Yang J. Bai B. Li G. Wang J. Li Z. . (2024). Non-destructive monitoring of peanut leaf area index by combing UAV spectral and textural characteristics. Remote Sens. 16, 2182. doi:10.3390/rs16122182. PMID: 30654563

[B54] Qiu J. Israel D. (1992). Diurnal starch accumulation and utilization in phosphorus-deficient soybean plants. Plant Physiol. 98, 316–323. doi:10.1104/pp.98.1.316. PMID: 16668630 PMC1080185

[B55] Rasmussen J. Ntakos G. Nielsen J. Svensgaard J. Poulsen R. Christensen S. (2016). Are vegetation indices derived from consumer-grade cameras mounted on UAVs sufficiently reliable for assessing experimental plots? Eur. J. Agron. 74, 75–92. doi:10.1016/j.eja.2015.11.026. PMID: 38826717

[B56] Rondeaux G. Steven M. Baret F. (1996). Optimization of soil-adjusted vegetation indices. Remote Sens. Environ. 55, 95–107. doi:10.1016/0034-4257(95)00186-7

[B57] Rouse J. W. Haas R. H. Deering D. W. Schell J. A. Harlan J. C. (1973). “ Monitoring the vernal advancement and retrogradation (green wave effect) of natural vegetation,” in Great plains corridor. (College Station, TX: Texas A&M University).

[B58] Sankaran S. Khot L. Zúñiga Espinoza C. Jarolmasjed S. Sathuvalli V. Vandemark G. . (2015). Low-altitude, high-resolution aerial imaging systems for row and field crop phenotyping: a review. Eur. J. Agron. 70, 112–123. doi:10.1016/j.eja.2015.07.004. PMID: 38826717

[B59] Sarsen G. Tang Q. Li Y. Bao L. Xu Y. Sun G. . (2026). A novel UAV-based fusion of spectral and textural features with canopy stratification for accurate estimation of vertical nitrogen distribution in cotton. Comput. Electron. Agric. 247, 111660. doi:10.1016/j.compag.2026.111660. PMID: 38826717

[B60] Shao M. An J. Liu B. Wu J. Zhang Q. Yao X. . (2025). Comprehensive assessment of wheat seedling growth status based on multimodal data. Sci. Agric. Sin. 58, 3857–3871. doi:10.3864/j.issn.0578-1752.2025.19.005. PMID: 41470978

[B61] Shaver T. Khosla R. Westfall D. (2006). “ Utilizing green normalized difference vegetation indices (GNDVI) for production level management zone delineation in irrigated corn,” in The 18th world congress of soil science. (Philadelphia, PA: 18th World Congress of Soil Science Organizing Committee. CD-ROM).

[B62] Shu Y. Huang G. Zhang Q. Peng S. Li Y. (2023). Reduction of photosynthesis under P deficiency is mainly caused by the decreased CO_2_ diffusional capacities in wheat (Triticum aestivum L.). Plant Physiol. Biochem. 198, 107680. doi:10.1016/j.plaphy.2023.107680. PMID: 37031546

[B63] Song D. Sun H. Ngumbi E. Kamruzzaman M. (2025). Multispectral image reconstruction from RGB image for maize growth status monitoring based on window-adaptive spatial-spectral attention transformer. Comput. Electron. Agric. 239, 111062. doi:10.1016/j.compag.2025.111062. PMID: 38826717

[B64] Sun B. Gao Y. Wu X. Ma H. Zheng C. Wang X. . (2019). The relative contributions of pH, organic anions, and phosphatase to rhizosphere soil phosphorus mobilization and crop phosphorus uptake in maize/alfalfa polyculture. Plant Soil 447, 117–133. doi:10.1007/s11104-019-04110-0. PMID: 30311153

[B65] Teshome F. Bayabil H. Hoogenboom G. Schaffer B. Singh A. Ampatzidis Y. (2023). Unmanned aerial vehicle (UAV) imaging and machine learning applications for plant phenotyping. Comput. Electron. Agric. 212, 108064. doi:10.1016/j.compag.2023.108064. PMID: 38826717

[B66] Thorp K. Wang G. Bronson K. Badaruddin M. Mon J. (2017). Hyperspectral data mining to identify relevant canopy spectral features for estimating durum wheat growth, nitrogen status, and grain yield. Comput. Electron. Agric. 136, 1–12. doi:10.1016/j.compag.2017.02.024. PMID: 38826717

[B67] Tucker C. (1979). Red and photographic infrared linear combinations for monitoring vegetation. Remote Sens. Environ. 8, 127–150. doi:10.1016/0034-4257(79)90013-0

[B68] Van D. Price K. (2015). Harmful algal bloom characterization at ultra-high spatial and temporal resolution using small unmanned aircraft systems. Toxins 7, 1065–1078. doi:10.3390/toxins7041065. PMID: 25826055 PMC4417955

[B69] Wang L. Bai Y. (2006). Research advance on plant nutrition diagnosis based on spectral theory. J. Plant Nutr. Fertil. 12, 902–912. doi:10.11674/zwyf.2006.0624

[B70] Wang Z. Lai N. Geng Q. Lyu C. Li Y. Xin H. . (2025). Research on phosphorus estimation model for winter wheat during critical growth stages based on hyperspectral data. Soil Fertil. Sci. China. 06, 237–245. doi:10.11838/sfsc.1673-6257.24553

[B71] Wei W. Wang D. Wang B. Tan Z. Liu Q. Xie J. (2025). Research on the inversion of moisture content in rapeseed silique peel based on hyperspectral fusion imaging. Spectrosc. Spectr. Anal. 45, 2863–2874. doi:10.3964/j.issn.1000-0593(2025)10-2863-12. PMID: 42102434

[B72] Woebbecke D. Meyer G. Bargen K. Mortensen D. (1995). Color indices for weed identification under various soil, residue, and lighting conditions. Trans. ASAE 38, 259–268. doi:10.13031/2013.27838

[B73] Xia Z. Zhang S. Wang Q. Zhang G. Fu Y. Lu H. (2021). Effects of root zone warming on maize seedling growth and photosynthetic characteristics under different phosphorus levels. Front. Plant Sci. 12, 746152. doi:10.3389/fpls.2021.746152. PMID: 34956256 PMC8695918

[B74] Xiao C. Zhang F. Mäkelä P. He J. Fan J. Jia Y. . (2025). An unmanned aerial vehicle-based cotton nitrogen nutrition index estimation model utilizing feature selection and machine learning. Comput. Electron. Agric. 238, 110798. doi:10.1016/j.compag.2025.110798. PMID: 38826717

[B75] Xie W. He P. Ma H. Lei F. Huang X. Fan G. . (2024). Effects of straw mulching from autumn fallow and phosphorus application on nitrogen uptake and utilization of winter wheat. Acta Agron. Sin. 50, 440–450. doi:10.3724/SP.J.1006.2024.31019

[B76] Xing Q. Gu Y. Gao Z. Ding S. (2008). “ Effects of N, P and K nutrition on photosynthesis and water use efficiency of winter wheat,” in Chin. J. Ecol. (Shenyang, China: Chinese Ecological Society), 355–360.

[B77] Xu T. Jin Z. Guo Z. Yang L. Bai J. Feng S. . (2022b). Simultaneous inversion method of nitrogen and phosphorus contents in rice leaves using CARS-RUN-ELM algorithm. Trans. Chin. Soc Agric. Eng. (Trans. CSAE) 38, 148–155. doi:10.11975/j.issn.1002-6819.2022.10.018

[B78] Xue H. Lou M. Li X. Wang F. Guo B. Guo D. . (2021). Effects of phosphorus application levels on growth and yield of winter wheat under different crops for rotation. Sci. Agric. Sin. 54, 3712–3725. doi:10.3864/j.issn.0578-1752.2021.17.013. PMID: 41470978

[B79] Yan R. Zhu L. Ran J. Yang W. Gong H. Liu J. (2023). Effects of phosphorus reduction measures on winter wheat yield and phosphorus uptake and utilization in Weibei dryland. J. Plant Nutr. Fertil. 29, 1265–1279. doi:10.11674/zwyf.2022675

[B80] Yao F. Zeng F. Sun J. Rao Z. Wang Z. (2025). Estimating vegetation chlorophyll content based on hyperspectral red edge skewness, kurtosis, and machine learning. Trans. Chin. Soc Agric. Mach. 56, 566–595. doi:10.6041/j.issn.1000-1298.2025.09.047

[B81] Yu Z. Wang X. Meng X. Zhang X. Wu D. Liu H. . (2019). SPAD prediction model of rice leaves considering the characteristics of water spectral absorption. Spectrosc. Spectr. Anal. 39, 2528–2532. doi:10.3964/j.issn.1000-0593(2019)08-2528-05. PMID: 42102434

[B82] Yuan Z. Tan X. (2010). Nonlinear screening indexes of drought resistance at rice seedling stage based on support vector machine. Acta Agron. Sin. 36, 1176–1182. doi:10.3724/SP.J.1006.2010.01176

[B83] Yun B. Xie T. Li H. Yue X. Lv M. Wang J. . (2024). Nitrogen nutrition estimation of maize based on UAV spectrum and texture information. Sci. Agric. Sin. 57, 3154–3170. doi:10.3864/j.issn.0578-1752.2024.16.005. PMID: 41470978

[B84] Zang S. Liu L. Gao Y. Wu K. He L. Duan J. . (2024). Classification and identification of nitrogen efficiency of wheat varieties based on UAV multi-temporal images. Sci. Agric. Sin. 57, 1687–1708. doi:10.3864/j.issn.0578-1752.2024.09.006. PMID: 41470978

[B85] Zhang X. Hu Y. Li X. Wang P. Guo S. Wang L. . (2025). Estimation of rice leaf nitrogen content using UAV-based spectral-texture fusion indices (STFIs) and two-stage feature selection. Remote Sens. 17, 2499. doi:10.3390/rs17142499. PMID: 30654563

[B86] Zhang C. Ren H. Dai X. Qin Q. Li J. Zhang T. . (2019). Spectral characteristics of copper-stressed vegetation leaves and further understanding of the copper stress vegetation index. Int. J. Remote Sens. 40, 4473–4488. doi:10.1080/01431161.2018.1563842. PMID: 37339054

[B87] Zhang P. Sun Y. Guo P. Yuan Z. Yang H. Bei M. . (2015). “ Study on predicting nitrogen content of rubber tree leaf by digital image analysis,” in Chin. J. Trop. Crops, vol. 36. (Haikou, China: Chinese Society of Tropical Crops), 2120–2124.

[B88] Zhang L. Sun B. Zhao D. Shan C. Wang G. Song C. . (2024a). Prediction of cotton FPAR and construction of defoliation spraying prescription map based on multi-source UAV images. Comput. Electron. Agric. 220, 108897. doi:10.1016/j.compag.2024.108897. PMID: 38826717

[B89] Zhang C. Xie Z. Shang J. Liu J. Dong T. Tang M. . (2022). Detecting winter canola (Brassica napus) phenological stages using an improved shape-model method based on time-series UAV spectral data. Crop J. 10, 1353–1362. doi:10.1016/j.cj.2022.03.001. PMID: 38826717

[B90] Zhang L. Yuan D. Fan Y. Yang R. Zhao M. Jiang J. . (2024b). Hyperspectral estimation of chlorophyll content in wheat under CO2 stress based on fractional order differentiation and continuous wavelet transforms. Remote Sens. 16, 3341. doi:10.3390/rs16173341. PMID: 30654563

[B91] Zhao J. Liang X. Kang X. Li Y. An W. (2024). Estimation of goji berry (Lycium barbarum L.) canopy water content based on optimal spectral indices. Sci. Hortic. 337, 113589. doi:10.1016/j.scienta.2024.113589. PMID: 38826717

[B92] Zhou W. He Y. Xu X. Li Y. (2024). Effects of different varieties of phosphorus fertilizer on phosphorus uptake and above-ground phosphorus form of winter wheat at seedling stage. Soil Fertil. Sci. China 11, 90–97. doi:10.11838/sfsc.1673-6257.24018

[B93] Zhou X. Yang M. Chen X. Ma L. Yin C. Qin S. . (2023). Estimation of cotton nitrogen content based on multi-angle hyperspectral data and machine learning models. Remote Sens. 15, 955. doi:10.3390/rs15040955. PMID: 30654563

[B94] Zhu Z. Wu Y. Ma J. Ji L. Liu B. Jin H. (2023). Response of winter wheat canopy spectra to chlorophyll changes under water stress based on unmanned aerial vehicle remote sensing. Spectrosc. Spectr. Anal. 43, 3524–3534. doi:10.3964/j.issn.1000-0593(2023)11-3524-11. PMID: 42102434

[B95] Zhu J. Zhao H. Lyu Y. Liang Z. Fu Y. Liang Y. . (2025). Evaluating freeze damage in winter wheat using vegetation index and texture-color features of unmanned aerial vehicle. Trans. Chin. Soc Agric. Eng. (Trans. CSAE) 41, 165–173. doi:10.11975/j.issn.1002-6819.202503144

